# Robust and High-Wettability Cellulose Separators with Molecule-Reassembled Nano-Cracked Structures for High-Performance Supercapacitors

**DOI:** 10.1007/s40820-025-01650-2

**Published:** 2025-02-19

**Authors:** Xiaoyu Wang, Wenqiu Zheng, Hui Zhao, Junying Li, Sheng Chen, Feng Xu

**Affiliations:** State Key Laboratory of Efficient Production of Forest Resources, Beijing Key Laboratory of Lignocellulosic Chemistry, Beijing Forestry University, Beijing, 100083 People’s Republic of China

**Keywords:** Superbase-derived ionic liquids, Molecular reassembly, Nano-structure, Cellulose separators, Supercapacitor

## Abstract

Separators in supercapacitors (SCs) frequently suffer from high resistance and the risk of short circuits due to inadequate electrolyte wettability, depressed mechanical properties, and insufficient thermal stability. Here, we develop a high-performance regenerated cellulose separator with nano-cracked structures for SCs via a binary solvent of superbase-derived ionic liquid and dimethylsulfoxide (DMSO). The unique nano-cracks with an average width of 7.45 nm arise from the acceleration of cellulose molecular reassembly by DMSO-regulated hydrogen bonding, which endows the separator with high porosity (70.2%) and excellent electrolyte retention (329%). The outstanding thermal stability (273 °C) and mechanical strength (70 MPa) enable the separator to maintain its structural integrity under high temperatures and external forces. With these benefits, the SC utilizing the cellulose separator enables a high specific capacitance of 93.6 F g^−1^ at 1.0 A g^−1^ and a remarkable capacitance retention of 99.5% after 10,000 cycles compared with the commercial NKK-MPF30AC and NKK-TF4030. The robust and high-wettability cellulose separator holds promise as a superior alternative to commercial separators for advanced SCs with enhanced performance and improved safety.

**Supplementary Information:**

The online version contains supplementary material available at 10.1007/s40820-025-01650-2.

## Highlights

- A robust and high-wettability cellulose separator with unique nano-cracked structures is constructed through hydrogen bond-driven reassembly of cellulose molecules using a green binary solvent.
- The nano-cracked structures endow the separator with high porosity (70.2%) and excellent electrolyte retention (329%).
- The supercapacitors with nano-cracked separators exhibit high specific capacitance (93.6 F g^−1^ at 1.0 A g^−1^) and a long cycling life (99.5% retention after 10,000 cycles).

**Supplementary Information:**

The online version contains supplementary material available at 10.1007/s40820-025-01650-2.

## Graphical abstract

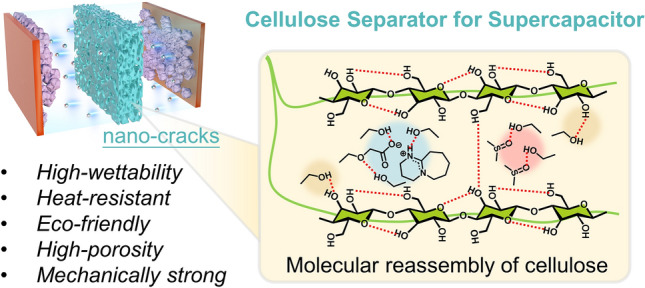

## Introduction

Supercapacitors (SCs) have been extensively employed across numerous sectors, particularly those that necessitate substantial discharge currents over brief spans, owing to their merits of swift charging/discharging abilities, outstanding cycle life, and high safety [1, 2]. The electrochemical performance of SCs is contingent upon several critical components, such as electrode, electrolyte, and separator. Numerous strategies have been proposed to augment the capacitance of SCs through the development or modification of electrode and electrolyte materials. Nonetheless, comparatively little focus has been directed toward designing separators that facilitate the rapid transport of ions, thereby reducing the internal resistance and improving the capacitance of SCs. As a vital component of SCs, separators effectively prevent electronic internal short circuits and ensure optimal interfacial connectivity [3–5]. Generally, separators for high-performance SCs in practical applications require excellent mechanical properties, sufficient thermal stability, uniform thickness, high porosity, outstanding wettability, and sufficient electrolyte storage capacity, while also featuring minimal ionic resistance [6, 7]. At present, polyolefin-based commercial separators, which are fabricated through extrusion casting or melt-blown methods, prevail in the field of SCs [8, 9]. However, the shrinkage at high temperatures, poor electrolyte wettability, low mechanical strength, and non-renewability of polyolefin-based separators reduce the safety, stability, and sustainability of SCs [10, 11]. Therefore, to address these challenges of commercial polyolefin-based separators, the development of high-performance separators based on sustainable materials is urgent.

Cellulose, the most abundant renewable polymer on the earth, exhibits exceptional biocompatibility, remarkable thermal stability, and high hydrophilicity, making it an ideal candidate for SC separators [12, 13]. The distinctive attributes of cellulose-based separators, including flexibility, absorbency, and biodegradability, can enhance the performance, environmental sustainability, and cost-effectiveness of SCs. Utilizing such separators, particularly in flexible SCs with high capacitance and energy density, such as those incorporating graphene or carbon nanotubes (CNTs), facilitates the development of advanced SCs for portable and wearable applications [14–16]. The commercial cellulose-based separators (e.g., NKK-TF4030) are usually made from ultrafine regenerated cellulose fibers with a diameter of 0.2–2.0 μm. These fibers are fabricated using *N*-methylmorpholine-*N*-oxide (NMMO)/H_2_O solvent and processed through a wet-laid nonwoven fabric paper-making method [2, 17]. It has been reported that SCs utilizing NKK-TF4030 separators demonstrated increased ionic conductivity, elevated specific capacitance, and superior rate capability in comparison with SCs utilizing polyolefin and nonwoven separators (e.g., Celgard 2400 and NKK-MPF30AC) [18–20]. To elevate the capacitance of SCs, separators are typically engineered with a porous architecture that promotes expeditious ion transport. The type of pore (micro-, meso-, or macropore) in the separators plays a pivotal role in determining the capacitance of SCs. Generally, macropores facilitate electrolyte infiltration, whereas mesopores and micropores abbreviate the ion diffusion pathways and diminish the collision between ions, thereby enhancing the electrochemical performance of SCs [21]. Nevertheless, the commercial cellulose-based separators have large pore sizes (0.31–0.45 μm), which would result in relatively high self-discharge rates and insufficient mechanical strength, thus limit the long-term application of SCs [22]. Therefore, cellulose-based separators necessitate high porosity to absorb and retain sufficient electrolyte, along with compatible pore size that decreases the self-discharge rate, ensuring superior ionic conductivity and excellent electrochemical performance.

The fabrication of regenerated cellulose materials depends on the effective dissolution and regeneration of cellulose [23, 24]. Currently, the prevalent cellulose solvents, such as NaOH/urea aqueous systems, molten salt hydrates, LiCl/*N*, *N*-dimethylacetamide (DMAc), and *N*-methylmorpholine-*N*-oxide (NMMO), encounter challenges related to suboptimal dissolution efficiency, environmental contamination, elevated cost, and safety hazards [25, 26]. Ionic liquids (ILs), as environmentally friendly cellulose solvents, have excellent cellulose solubility, chemical stability, recyclability, and low vapor pressure [26–29]. Numerous cellulose-based separators with superior tensile stress, thermal stability, and electrolyte uptake have been developed using imidazolium-based ILs, such as 1-allyl-3-methylimidazolium chloride ([Amim]Cl) and 1-butyl-3-methylimidazolium chloride ([Bmim]Cl) [30–32]. However, the high viscosity of ILs necessitates elevated temperatures and extended mixing times for cellulose dissolution [29]. Besides, cellulose films regenerated from high-viscosity IL/cellulose solutions by the physical phase inversion utilizing anti-solvents exhibit non-porous structures, which hinder the rapid ion transport in SCs [4, 33]. The mesoporous structures of these separators commonly arise from complex and unsustainable methods during cellulose regeneration, including the use of porous templates (e.g., polytetrafluoroethylene) [20, 34], incorporation of nanoparticles (e.g., calcium carbonate and chitosan) followed by acid leaching [18, 35], and production of gases through high-temperature decomposition of substances (e.g., (NH_4_)_2_CO_3_) [36]. To date, no technique enables rapid cellulose dissolution while constructing cellulose films with suitable porous structures by regulating the regeneration process. Consequently, it remains challenging to develop cellulose-based SC separators with high porosity, exceptional electrolyte wettability, superior thermal stability, and outstanding mechanical strength using an efficient, sustainable, and eco-friendly approach [2, 35].

**Fig. 1:**
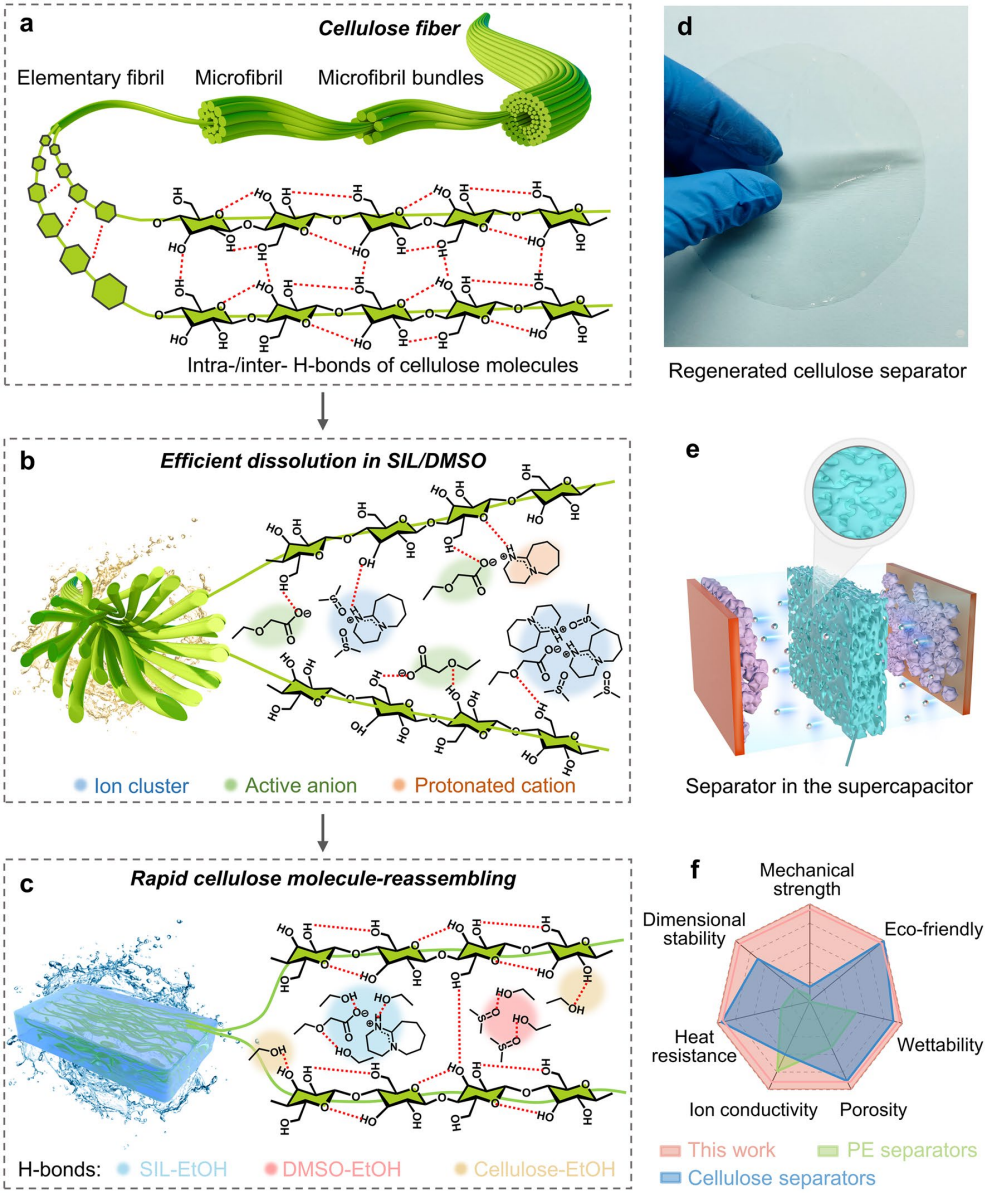
Fabrication mechanism and advantages of the nano-cracked cellulose separator. **a**–**c** Schematic illustration of the preparation of regenerated cellulose separator for supercapacitor through efficient dissolution and regeneration of cellulose in a novel binary system (SIL/DMSO). **d** Optical photo of regenerated cellulose separator (SORC film). **e** Schematic illustration of supercapacitor assembled using SORC film as the separator. The partial magnification shows the microscopic defect structure (nano-cracks) of the separator. **f** Radar chart demonstrating the advantages of the SORC film as the supercapacitor separator

Here, we develop a simple and effective method to prepare the regenerated cellulose separator with functional defect structure. This process employs a novel binary system of superbase-derived ionic liquid (SIL) and dimethylsulfoxide (DMSO) to enable efficient dissolution and rapid regeneration of cellulose, facilitating the formation of cellulose molecule-reassembled nano-cracked films for high-performance SC separators (Fig. 1a–d). The dissolution and regeneration processes are monitored by polarized light microscopy (PLM) and *in situ* attenuated total reflection Fourier-transform infrared (FTIR) spectroscopy to reveal the role of DMSO in disrupting and reorganizing inter- and intramolecular hydrogen bonds of cellulose. This pore-formation technique employed in this work is more straightforward and effective than the dissolution–regeneration method used for regenerated cellulose separators. Specifically, the DMSO involved in the dissolution process modulates the reassembly of cellulose hydrogen bonds during regeneration, creating nano-cracked structures that facilitate ion transport (Fig. 1e). Compared with commercial polyolefin-based and cellulose-based separators, the regenerated cellulose separator fabricated from SIL/DMSO (SORC) exhibits remarkable properties, including robust mechanical strength, adequate thermal stability, exceptional dimensional stability, high porosity and electrolyte wettability, and superior ionic conductivity (Fig. 1f). Furthermore, SCs are assembled with potassium hydroxide (KOH) as the electrolyte, SORC film as the separator, and activated carbon as the electrodes. Electrochemical performances of SCs are investigated and compared with those based on the regenerated cellulose separator fabricated from pure SIL (SRC) and the commercial separators (NKK-MPF30AC and NKK-TF4030).

## Experimental Section

### Materials

Dissolving wood pulp with the polymerization degree of 526 was provided by Tembec Inc. in Canada. The pulp was dried at 60 °C for 24 h under vacuum conditions before use. 1,8-diazabicyclo [5.4.0] undec-7-ene (DBU) (99%), ethoxyacetic acid (CH_3_CH_2_OCH_2_COOH) (98%), n-Hexane (99%), DMSO (99.8%), and KOH were purchased from Shanghai Macklin Biochemical Co., Ltd. NKK-MPF30AC, NKK-TF4030, activated carbon, acetylene black, and 60 wt% polytetrafluoroethylene (PTFE) emulsion were purchased from Dongguan Canrud New Energy Technique Co., Ltd. All chemicals and reagents used as received without further purification.

### Preparation of Cellulose/SIL/DMSO Solutions and Regenerated Cellulose Films

The SIL was synthesized by acid–base neutralization described in our previous work [26]. SIL was mixed with DMSO in different mass ratios. The appropriate amount of the dissolving wood pulp was immersed into a 50 mL flask containing SIL/DMSO solvent for 10 min to swell fibers. The cellulose/SIL/DMSO solution with the cellulose concentration of 4 wt% was prepared by stirring magnetically at 80 °C until the pulp was completely dissolved, as observed by polarized light microscopy (Olympus Corporation Tokyo 163-0914, Japan). The preparation of cellulose/SIL solution followed the similar procedures. In addition, the fabrication of RC films adhered to our previously reported methodology. Briefly, a 15 mL 4 wt% cellulose solution was cast into a glass petri dish with a diameter of 9 cm, and the resulting RC film was obtained through coagulation with ethanol and washing with deionized water. All RC films were subjected to vacuum drying in an oven at 60 °C for 12 h. The film fabricated from the cellulose/SIL solution was named SRC film, while the film fabricated from the cellulose/SIL/DMSO solution was named SORC film.

### Assembly of Supercapacitors

The working electrode prepared with 80 wt% activated carbon, 10 wt% acetylene black, and 10 wt% PTFE was dried in an oven at 60 °C for 12 h before use. The areal mass loading of activated carbon for the working electrode is around 2.4 mg cm^−2^. The electrodes and RC films were immersed into a 6 M KOH aqueous solution for 48 h, and excess electrolyte was removed from the surface using absorbent tissue paper. The cellulose-based supercapacitor was assembled in a CR2432-type coin cell configuration of electrode//RC film/KOH//electrode. Similar supercapacitors containing commercialized NKK-MPF30AC or NKK-TF4030 as separators were also prepared for comparison.

### Computational Methods

The geometry optimizations of the SIL ([DBUH][CH_3_CH_2_OCH_2_COO]), DMSO, cellobiose (cellulose model), and ethanol were performed using the density functional theory (DFT) method with the B3LYP functional and the def2SVP basis set as implemented in the Gaussian 09 package [37–39]. Then, the structures of ethanol-SIL, ethanol-cellobiose, ethanol-DMSO, SIL-DMSO, and SIL-DMSO-cellobiose were optimized on the same basis set, and the gd3bj term was used for the dispersion correction [40]. To get more accurate data in the energy, single-point calculations were performed with 3-zeta basis set in the def2TZVP [39, 41]. The binding energy (*ΔE*_binding_) between the different components in the complexes is defined based on Eq. (1):

1 $$\Delta E_{\text{binding}} = \Delta E_{\text{complex}} - \Delta E_{\text{compound1}} - \Delta E_{\text{compound2}} - \Delta E_{\text{compound x}}$$

where *ΔE*_compound_ represents the different components in the complex, such as cation and anion in SIL, cellobiose, and DMSO. In addition, the independent gradient model based on Hirshfeld partition (IGMH) of the complex structures was calculated by the Multiwfn program, and the corresponding renderings were performed with the VMD 1.9.3 [42, 43].

### Characterization Methods

The Supplementary Information detailed characterization methods for the physical properties of solvents and cellulose solutions, the regeneration process of cellulose, the structure and properties of separators, and the electrochemical performance of SCs.

## Results and Discussion

### Fabrication of the Cellulose Molecule-Reassembled Nano-Cracked Film

**Fig. 2:**
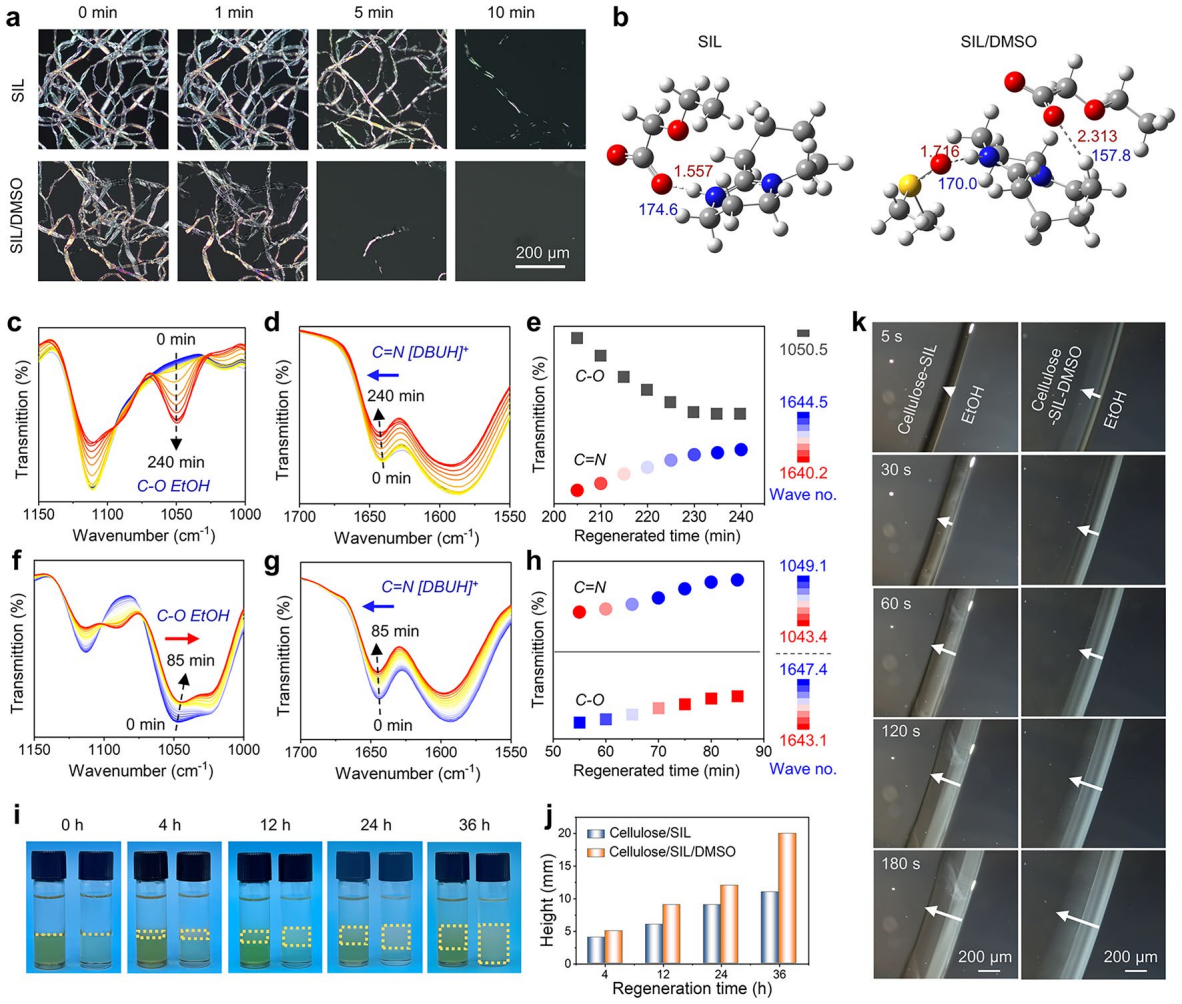
Cellulose dissolution and regeneration processes. **a** PLM images of dissolution process of DWP fibers in SIL/DMSO solvent. **b** Optimized structures and hydrogen bonds of SIL and SIL-DMSO. Time-dependent FTIR spectra of *in situ* regeneration from **c-e** cellulose/SIL solution in the time 0–240 min and **f**–**h** cellulose/SIL/DMSO solution in the time 0–85 min using EtOH as a coagulation bath, displaying at 5 min interval. **i** Digital photographs of cellulose regeneration at different time intervals. Left: cellulose/SIL solution in EtOH; right: cellulose/SIL/DMSO solution in EtOH bath. The yellow lines represent the regenerated cellulose gels. **j** Heights of the regenerated cellulose gels at different time intervals. **k** Advancement of phase inversion front detected by PLM for cellulose solutions. The arrow points in the direction of EtOH diffusion into the cellulose solution

Cellulose was effectively dissolved in a low-viscosity, high-efficiency SIL/DMSO solvent with the optimized mass ratio of SIL to DMSO (7:3) (Fig. S1). As illustrated in PLM images (Fig. 2a and Movies S1, S2), a large clump of undissolved pulp fibers in pure SIL was detected after heating at 80 °C for 5 min, whereas few undissolved fibers were observed in SIL/DMSO. The accelerated dissolution of cellulose in SIL/DMSO was mainly attributed to the presence of DMSO that acted as a hydrogen bonding acceptor interacting with SIL cations ([DBUH^+^]) through the strong hydrogen bonds (Fig. 2b) (Fig. S2 and Table S1). This may contribute to the increase of free active anion ([CH_3_CH_2_OCH_2_COO]^−^) and the formation of small ion clusters in SIL/DMSO solvent, facilitating the disruption of hydrogen bonds of cellulose (Fig. S3) [44, 45]. The resulting low-viscosity homogeneous cellulose/SIL/DMSO solution exhibited excellent spreadability and processability at ambient temperature, coupled with outstanding stability at high shear rates (Fig. S4). Hence, the SIL/DMSO solvent served as a straightforward and provided a simple and efficient means of preparing a low-viscosity and stable cellulose solution used for the casting film.

Cellulose regeneration is typically realized through a physical sol–gel process that utilizes anti-solvents to reassemble hydrogen bonding network of cellulose [23, 32, 46]. To investigate the effect of DMSO on molecular reassembly of cellulose, time-dependent ATR-FTIR was employed to monitor the diffusion processes of SIL and ethanol (EtOH) in situ. At the outset, solely the characteristic peaks of SIL were presented in the spectra, as the thickness of the cellulose solution droplet exceeded the depth of the evanescent wave penetration (~ 1 μm) (Fig. S5) [47]. As EtOH diffused into the evanescent field, a characteristic absorption peak at 1050 cm^−1^ attributed to C–O stretching vibration of EtOH was detected. The peak intensity increased as the diffusion time prolonged, ultimately reaching a balanced state (Fig. 2c, e). However, the cellulose/SIL/DMSO solution without EtOH showed a strong absorption peak at around 1050 cm^−1^ due to the stretching vibration of the S = O bond in DMSO (Fig. 2f). Interestingly, this peak intensity gradually decreased and exhibited a significant red shift (from 1049 to 1043 cm^−1^) as the regeneration proceeded (Fig. 2h), implying a strong interaction between EtOH and DMSO [48]. In addition, the characteristic peak of the C=N stretching vibration of [DBUH]^+^ in the SIL at 1640 cm^−1^ shifted to a higher wavenumber with a gradual decrease in intensity as the EtOH diffused into the cellulose solutions (Fig. 2d, e, g, h). Notably, this characteristic peak detected in the cellulose/SIL solution from the beginning to a constant position required a longer time (230 min) than that detected in the cellulose/SIL/DMSO solution (75 min). This may be attributed to the low viscosity of the cellulose/SIL/DMSO solution and the interaction of DMSO with EtOH, which improved the mass transfer efficiency of EtOH and accelerated the disruption of hydrogen bonds between SIL and cellulose [49, 50].

The digital photographs taken at different time intervals of cellulose solutions coagulated in EtOH provided additional support for the findings above (Fig. 2i, j). Apparently, cellulose/SIL/DMSO solution was completely precipitated after EtOH diffusion for 36 h, while only 55% cellulose/SIL solution was precipitated under the same duration. Moreover, the phase inversion processes of cellulose solutions were monitored under the PLM by the advancement of the phase inversion front in time (Movies S3, S4). Figure 2k displays the front at *t* = 5, 30, 60, 120, and 180 s for the films made from cellulose solutions and coagulated in EtOH. As compared with cellulose/SIL solution, the cellulose/SIL/DMSO solution exhibited a higher phase inversion rate and thus the front proceeded more rapidly. This result was consistent with the above *in situ* FTIR analysis. Notably, substantial solvent diffusion trajectories (at 180 s) were observed in the cellulose/SIL/DMSO solution during phase inversion, implying the formation of loose structure of regenerated cellulose.

**Fig. 3:**
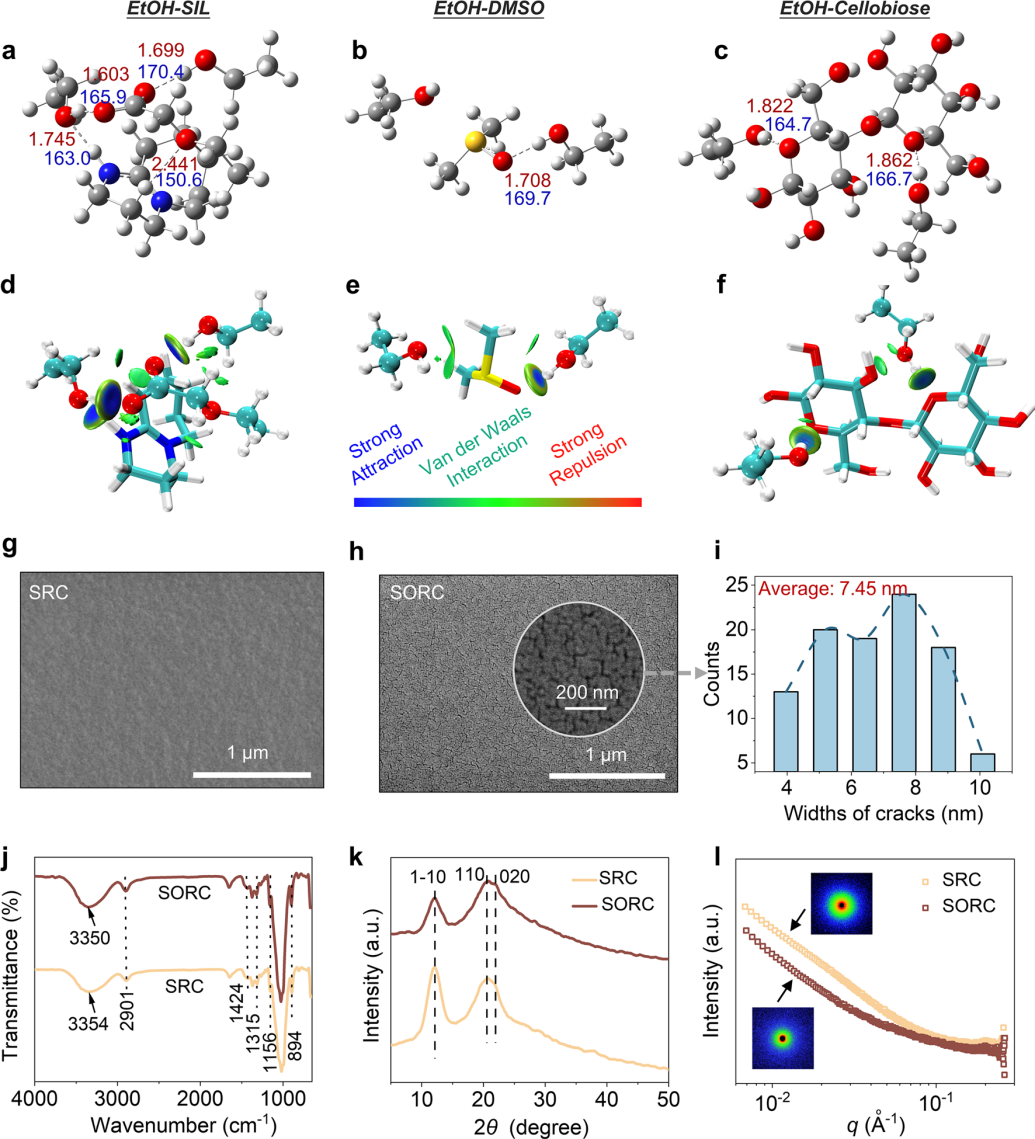
Interaction and structure characterizations. **a**–**c** Optimized structures of EtOH-SIL, EtOH-DMSO, and EtOH-cellobiose. Carbon: dark gray, Hydrogen: light gray, Oxygen: red, Nitrogen: blue, Sulfur: yellow. **d**–**f** IGMH surface analysis of EtOH-SIL, EtOH-DMSO, and EtOH-cellobiose (isovalue = 0.01 a.u.). Distinctions between interaction components are made with bat and stick models, with blue indicating strong interactions, green indicating van der Waals interactions, and red indicating site resistance. Surface SEM images of the **g** SRC film and **h** SORC film. **i** Width distribution of cracks on the surface of SORC. **j** FTIR spectra, **k** WAXS patterns, and **l** SAXS patterns of SRC film and SORC film. The insert images represent the 2D-SAXS patterns

The reorganization of the hydrogen bonding network in the cellulose regeneration is a vital step toward obtaining satisfactory material properties [51]. To delve into the formation mechanism of cellulose regenerated from cellulose/SIL/DMSO solution using EtOH as the anti-solvent, DFT calculations were carried out. The structure of EtOH is generally considered as a dynamic three-dimensional hydrogen bond network, with EtOH molecules functioning as both donors and acceptors of hydrogen bonds [52]. As shown in Fig. 3a–c, EtOH interacted with SIL, DMSO, and cellulose (with cellobiose as a model compound) through hydrogen bonds. The strength of hydrogen bonding interactions was in the following order: EtOH-SIL (− 19.55 kcal mol^−1^) > EtOH-cellobiose (− 16.23 kcal mol^−1^) > EtOH-DMSO (− 7.72 kcal mol^−1^) (Table S2). These interactions are graphically depicted by the independent gradient model based on Hirshfeld partition (IGMH) [53], in which van der Waals forces between EtOH and the components are also observed (Fig. 3d–f). Hence, the regeneration process commenced as the cellulose/SIL/DMSO solution contacted with EtOH. This process was directly influenced by the competing hydrogen bonding interactions among cellulose, SIL, DMSO, and EtOH, which depended on the hydrogen bond donor/acceptor property of the solvent [25, 54]. Cellulose interacted with SIL through hydrogen bonds when cellulose was dissolved in SIL/DMSO solvent. DMSO acted as a weak acceptor of hydrogen bonds, which hardly affected the strength of interactions between cellulose and SIL. During the regeneration process of cellulose, EtOH preferentially formed hydrogen bonds with SIL anions, leading to the reorganization of both inter- and intramolecular hydrogen bonds in cellulose. DMSO played a crucial role in optimizing hydrogen bond network in this process, allowing for a favorable structure for further applications (Fig. S6). Consequently, the strong hydrogen bonds between EtOH and SIL were the main driving force for cellulose molecular reassembly. Meanwhile, DMSO significantly reduced the probability of both intra- and inter-molecular hydrogen bonds within cellulose, resulting in regenerated cellulose with loose structures.

According to the investigations above, flexible and transparent renewable cellulose films were fabricated with SIL and SIL/DMSO as solvents (Fig. S7). The surface morphology of the SRC film was dense and devoid of visible pores or cracks (Figs. 3g and S8a). In contrast, the SORC film exhibited pronounced nano-crack structures with an average width of 7.45 nm due to the loose structure of cellulose that regenerated from the cellulose/SIL/DMSO solution (Figs. 3h, i and S8b). This structure endowed the film with high porosity and efficient ion transport, which potentially diminished the diffusion pathway for ions mitigating ionic collisions, showing significant potential for utilization as separators of energy storage devices. Moreover, the uniform distribution of nano-cracks within the film may be conducive to fine filtration and substance separation, including applications in water treatment, gas purification, and biomolecular isolation [55]. The chemical and crystalline structure of RC films was further characterized using FTIR, wide-angle X-ray scattering (WAXS), and small-angle X-ray scattering (SAXS). The FTIR spectrum revealed that bands at 2901, 1424, and 894 cm^−1^ were attributed to C–H stretching, HCH and OCH bending vibrations, and *β*-glycosidic linkage, respectively (Fig. 3j) [24]. No noticeable differences were observed in RC films, demonstrating that DMSO did not affect the chemical structure of cellulose in dissolution and regeneration. The WAXS patterns of RC films displayed diffraction peaks at around 12.5°, 20.5°, and 22.0°, which were assigned to the hydrophilic (1–10) and hydrophobic (110) and (020) planes, respectively, representing the crystalline structure of cellulose II (Fig. 3k) [56]. Furthermore, the circular symmetry observed in the 2D-SAXS patterns of RC films suggested that the morphologies of regenerated cellulose possessed essentially isotropy (Fig. 3l). The *q* range of 0.01–0.06 Å^−1^ corresponds to the microfibril structure, characterized by an aggregate size ranging from 10.5 to 62.8 nm [57]. The SAXS intensity of the SORC film within this *q* range was decreased in comparison with the SRC film, indicating the formation of smaller aggregates in the SORC film, consequently leading to its nano-cracked structures.

### Application Potential of the SORC Film as a Supercapacitor Separator

**Fig. 4:**
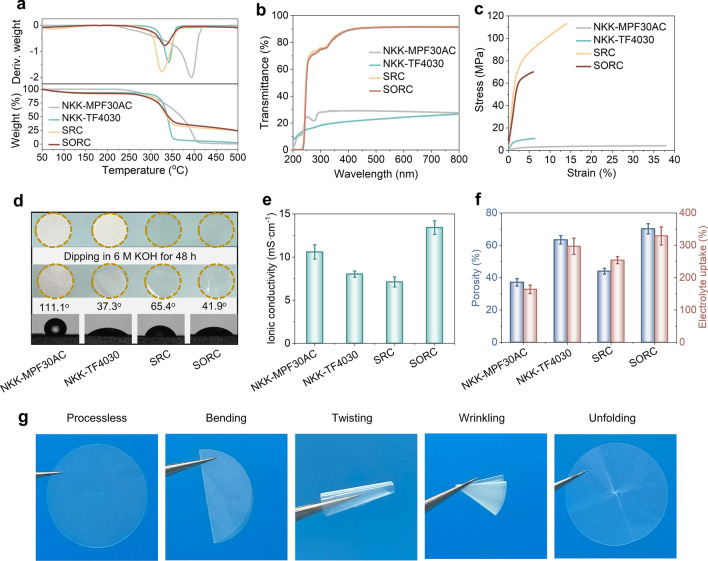
Physical properties of the RC films. NKK-MPF30AC and NKK-TF4030 were also investigated for comparison. **a** Thermogravimetric (TG) and derivative TG (DTG) curves. **b** Light transmittance. **c** Tensile stress–strain curves. **d** Static contact angles for a droplet of 6 M KOH solution, and absorption-swelling characteristics. **e** Ionic conductivity. **f** Porosity and electrolyte uptake. **g** Optical photographs of the SORC separator before and after bending, twisting, and wrinkling

Thermal stability of polymer separators is an essential aspect to assess the safety of energy storage devices [58]. The maximum thermal degradation temperature of the SORC film was approximately 330 °C (Fig. 4a), which was comparable to that of the SRC film and NKK-TF4030 (325–340 °C), but lower than that of NKK-MPF30AC (392 °C). Although inferior to NKK-MPF30AC in this regard, the thermal stability of the SORC film exceeded most polyolefin-based separators, such as polyethylene (PE) and poly(aryl ether sulfone) [59]. Significantly, the thickness of separators employed in commercial and high-performance energy storage devices is typically confined to a range of 20–100 μm, a constraint that is imperative for ensuring both operational safety and optimal energy storage performance. The obtained SORC film, with a thickness of 27 μm, was comparable to the previously reported advanced cellulose separators [60, 61]. Moreover, this film exhibited a high light transmittance of 91.5% (Fig. 4b) and a low haze of 20.2% at 800 nm (Fig. S9). The tensile strength and elongation at break of the SORC film were 69.93 MPa and 5.95%, respectively (Fig. 4c). These values substantially exceeded the standard requirements for the safe operation of stacked SCs [62]. In comparison, the strength value of the SORC film surpassed those of NKK-MPF30AC (4.21 MPa) and NKK-TF4030 (10.57 MPa). This advantage played an essential role in maintaining structural integrity and preventing separator rupture, thereby enhancing the safety of SCs.

The electrolyte wettability of separators plays a crucial role in the assembly process and electrochemical performance of SCs [2, 63]. The static contact angle between the SORC film and 6 M KOH solution was 41.94°, which gradually decreased over time, eventually reaching zero after five minutes (Figs. 4d and S10). Notably, the SORC film exhibited excellent dimensional stability following 48 h of immersion in a 6 M KOH solution, with no significant shrinkage or expansion observed. Benefiting from the formation of uniformly distributed nano-cracked structures and the inherent excellent hydrophilic ability, the SORC film possessed superior aqueous electrolyte wettability, facilitating the efficiency of ion transport in the separators, thereby contributing to a higher ion conductivity (13.41 mS cm^−1^) (Fig. 4e). This value was noticeably higher than that of commercial NKK-MPF30AC (10.59 mS cm^−1^) and NKK-TF4030 (8.02 mS cm^−1^). Apart from the electrolyte wettability, the ionic conductivity of a separator significantly relies on its porosity and electrolyte retention [64]. Generally, high porosity allows for numerous ion diffusion pathways and sufficient space for electrolyte absorption. The SORC film demonstrated a porosity of 70.2% and an electrolyte uptake of 329%, outperforming dense cellulose film (SRC) (44%, 254%), nonwoven polypropylene film (NKK-MPF30AC) (37.1%, 164%), and cellulosic paper (NKK-TF4030) (63.4%, 297%) (Fig. 4f). During the assembly, storage, and transportation of SCs, deformation of the separator resulting from various stress collisions is commonly inevitable [65]. Hence, the mechanical flexibility is considered as a crucial property for the separator. As shown in Fig. 4g, after subjecting the SORC films to demanding tests involving bending, and wrinkling, they consistently maintained their flatness, demonstrating exceptional flexibility. In summary, the SORC film, possessing unique nano-cracked structures, demonstrates sufficient thermal stability, high ionic conductivity, exceptional electrolyte wettability, and superior mechanical strength. This renders it a promising contender for utilization as a separator in SCs and batteries.

### Electrochemical Performance of Supercapacitor with the SORC Separator

**Fig. 5:**
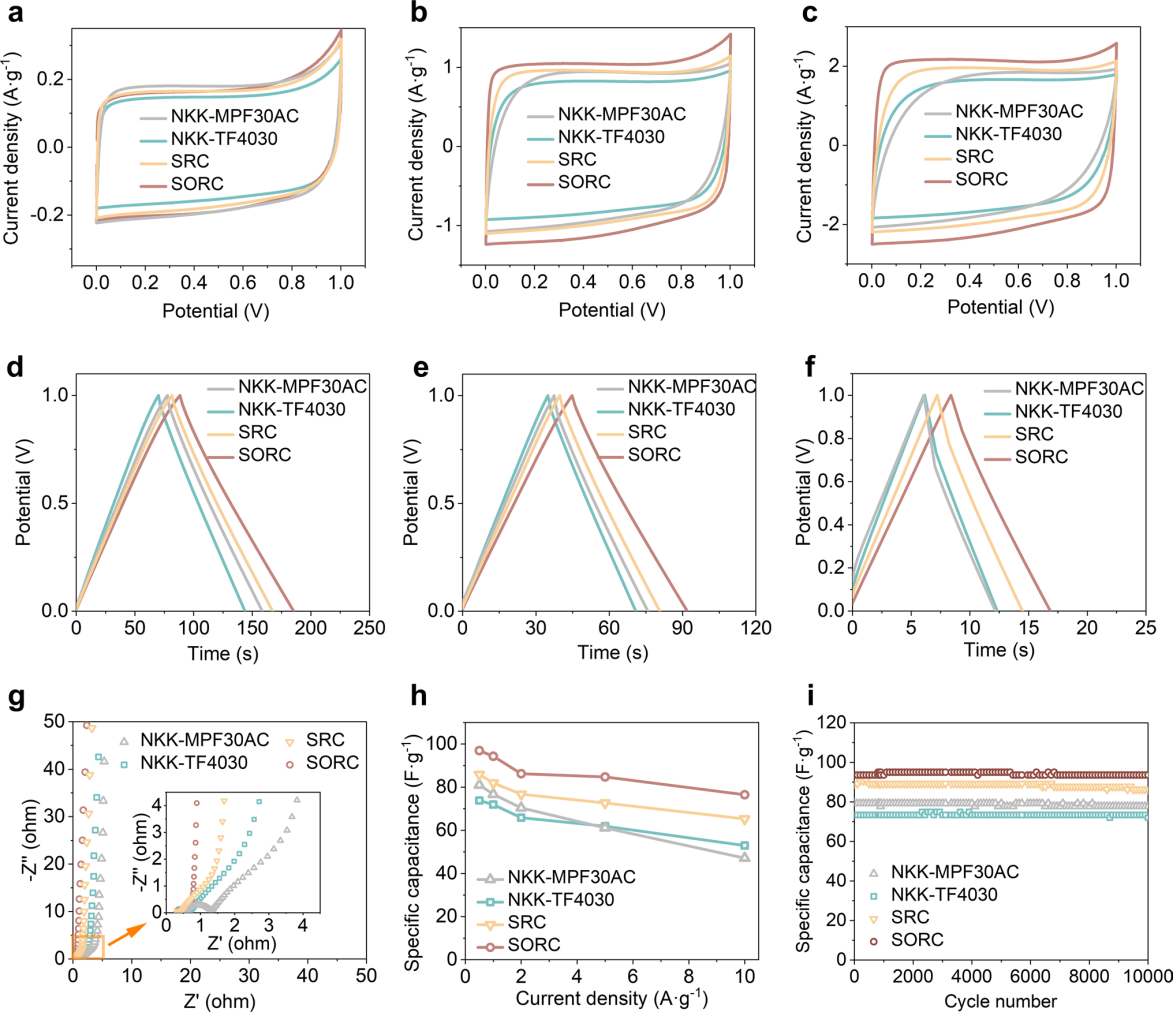
Electrochemical properties of the SC using SORC film as separator. Similar SCs containing NKK-MPF30AC, NKK-TF4030, and SRC as separators were also tested for comparison. **a**–**c** CV curves measured at scan rates of 10, 50, and 100 mV s^−1^, respectively. **d**–**f** GCD profiles at current densities of 0.25, 0.5, and 2.5 A g^−1^, respectively. **g** Nyquist plots. **h** Specific capacitances measured at different current densities. **i** Cycling performance measured at a current density of 1.0 A g^−1^

SCs were assembled in coin cells configuration of electrode//separator/KOH//electrode (Fig. S11a). These SCs produced reversible electrochemical electric double-layer capacitance at the electrode–electrolyte interface for energy storage, in which charges accumulated on the surface of the activated carbon electrodes and oppositely charged ions were arranged on the KOH electrolyte side (Fig. S11b). The high-performance cellulose separator, a pivotal component, served to avert direct contact between the electrodes while facilitating effective ion transport. To evaluate electrochemical performance of SCs, cyclic voltammetry (CV) curves were obtained at scan rates of 10, 50, and 100 mV s^−1^ (Fig. 5a–c). The CV curves exhibited similar profiles across a broad potential range of 0–1.0 V. Notably, the integrated areas of the CV curves for the SCs containing SORC separator exceeded those of the SCs containing NKK-MPF30AC, NKK-TF4030, and SRC separators at high scan rates. This result indicated that the SC with the SORC separator demonstrated superior equivalent SC behavior and a higher specific capacitance. Galvanostatic charge/discharge (GCD) profiles were measured at various current densities, as depicted in Fig. 5d–f. The SC with SORC separator demonstrated a prolonged discharge duration and a diminished voltage drop compared to the other three SCs. Specifically, the discharge time of the SC with SORC separator reached 8.5 s at 2.5 A g^−1^, which was 1.4 times better than that of the SC with NKK-MPF30AC separator (6.1 s) or with NKK-TF4030 separator (6.2 s), and 1.2 times better than that of the SC with SRC separator (7.3 s). Moreover, the Nyquist plots were obtained using the alternating current impedance technique across a frequency range of 0.01–100 kHz (Fig. 5g). The intercept with the real axis at high frequencies identified the series resistance of SCs. Remarkably, the SC with the SORC separator exhibited an equivalent series resistance of 0.20 Ω, which was smaller than that observed for the SC with SRC separator (0.23 Ω), the SC with NKK-TF4030 separator (0.24 Ω), and the SC with NKK-MPF30AC separator (0.54 Ω).

As shown in Fig. 5h, SCs possessed favorable specific capacitances across a current density range of 0.5–10.0 A g^−1^. Specifically, the specific capacitance values for the SC with SORC separator were 1.1–1.3 times better than those of the SCs with SRC, NKK-MPF30AC, and NKK-TF4030 separators. The capacitance value of 93.6 F g^−1^ at 1.0 A g^−1^ for the SC, which incorporated a SORC separator, activated carbon electrodes, and a 6 M KOH electrolyte, was notably inferior to those reported for SCs utilizing electrodes with optimized pore structures, improved electrical conductivity, and increased specific surface areas, as well as electrolytes with high ionic conductivity (Table S3). Nevertheless, this value surpassed the previously reported values of 62.5 F g^−1^ at 0.5 A g^−1^ for the SC with a porous regenerated cellulose film and 64.8 F g^−1^ at 0.25 A g^−1^ for the SC with a cellulose nano-fibril (CNF) membrane in a 6 M KOH electrolyte [4, 66]. Impressively, the SC with a SORC separator exhibited a remarkable capacitance retention of 99.5% even after 10,000 charge/discharge cycles at a current density of 1.0 A g^−1^. This performance surpassed that of the commercial separators NKK-MPF30AC (77.6 F g^−1^, 95.7%) and NKK-TF4030 (72 F g^−1^, 97%) (Fig. 5i). Furthermore, the SC demonstrated a coulombic efficiency approaching 100%, suggesting its exceptional long-term cyclic stability (Fig. S12). Besides, evaluating the electrochemical performance of SCs requires considering crucial parameters like energy density and power density. As shown in Fig. S13, the energy density of the SC with the SORC separator was higher than that of the SCs with the previously reported commercial and porous cellulose-based separators [20]. These results unequivocally indicated that the implementation of SORC film as a separator in the SCs presented exceptional electrochemical performance, surpassing that of SCs employing SRC film and commercially available separators. This superiority can be primarily attributed to the abundant active hydrogen groups, excellent mechanical properties, nano-cracked structures, and high porosity exhibited by the SORC separator, which facilitated increased electrolyte retention and ion transport pathways, thereby enhancing ionic conductivity and cycling stability.

### Lifetime Stability and Heat Resistance of Supercapacitor with the SORC Separator

**Fig. 6:**
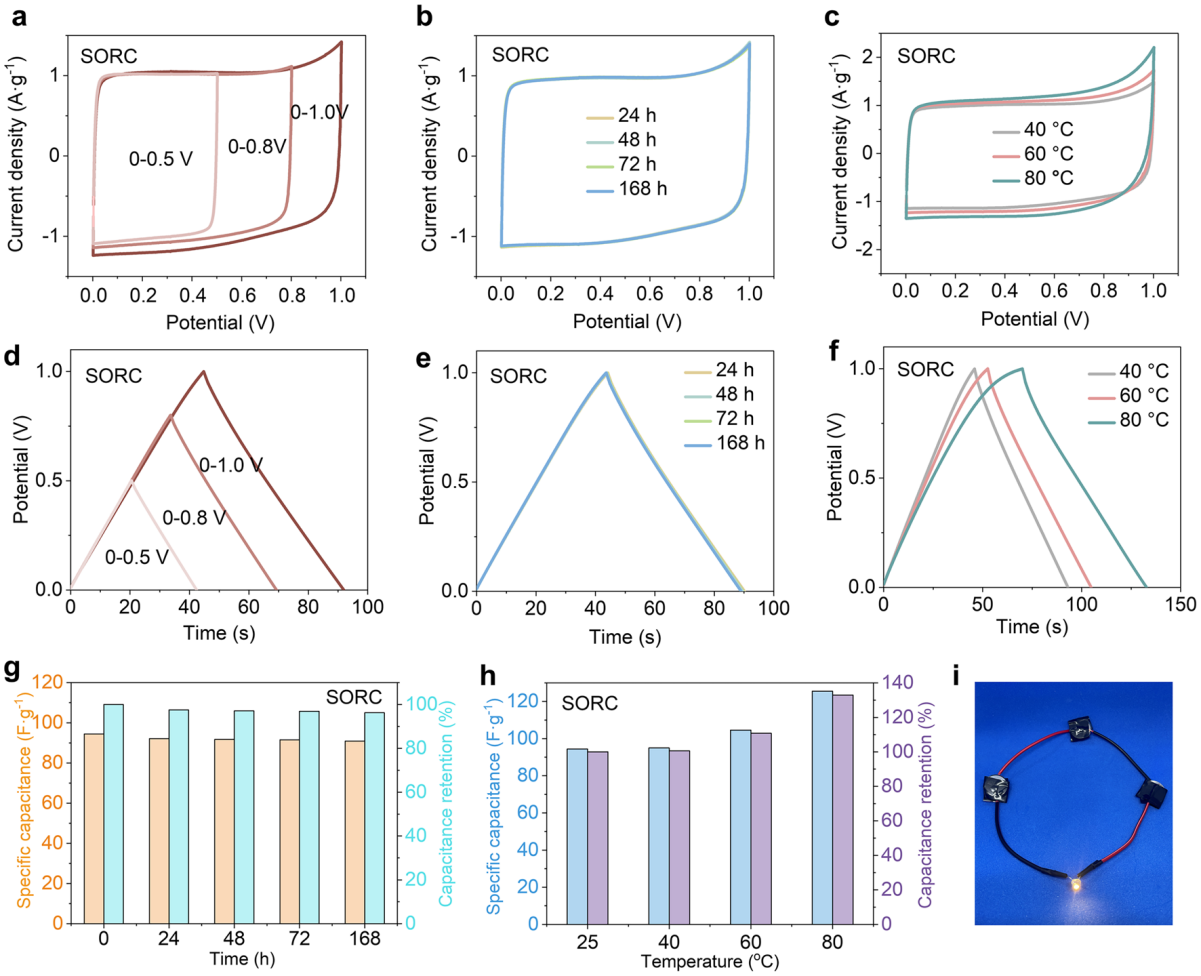
Lifetime stability and heat resistance of the SC using SORC film as the separator. **a**–**c** CV curves at a scan rate of 50 mV s^−1^. **d**–**f** GCD profiles at a current density of 1.0 A g^−1^. **g, h** Specific capacitances and capacitance retention. **i** Photograph of three SCs with SORC separators turning on a 3.0 V LED lamp in series

The lifetime stability of the SC with SORC separator was evaluated by exploring its electrochemical performance throughout various scan potential windows and at different time intervals (Fig. 6). For comparison, the SC with a commercial NKK-MPF30AC separator was also tested (Fig. S14). The CV and GCD curves maintained their quasi-rectangular and symmetrical triangular shapes within the scan potential windows of 0–0.5, 0–0.8, and 0–1.0 V, respectively (Fig. 6a, d). This result indicated that the SC with SORC separator exhibited adaptability across diverse potential windows. Moreover, the shapes of CV curves at 50 mV s^−1^ and GCD curves at 1.0 A g^−1^ remained stable over time (Fig. 6b, e). The GCD curves demonstrated significant overlap, and no noticeable decline in capacitance retention was observed (Fig. 6g). Remarkably, the specific capacitance of the SC with SORC separator consistently exceeded that of the SC with NKK-MPF30AC separator after different time intervals (Figs. 6g and S15). The capacitance retention of the SC with SORC separator reached an impressive value of 96.3% after 168 h, affirming its long lifetime stability.

To further investigate the potential of the SC with SORC separator operating under elevated thermal conditions, measurements of the electrochemical performance across a temperature range of 40 to 80 °C are presented in Fig. 6c, f. The CV curves for SCs with SORC and NKK-MPF30AC separators, using a scan potential range of 0–1.0 V, exhibited nearly rectangular shapes at various temperatures (Figs. 6c and S16a). This suggested that the charge and discharge processes were predominantly reversible and kinetically facile, even when operated at elevated temperatures. The superior capacitive behavior and electrochemical reversibility at different temperatures were further confirmed by GCD curves, which displayed symmetric triangle shapes (Figs. 6f and S16b). Through examination of the GCD profiles, it was ascertained that the specific capacitance values of the SC with SORC separator were 95.0, 104.6, 125.5, and 150.3 F g^−1^ at 40, 60, 80, and 100 °C, respectively (Fig. 6h). These values were significantly higher than those obtained with the SC using the NKK-MPF30AC separator, which achieved specific capacitances of 78.9, 86.3, 103.3, and 113.1 F g^−1^ at the corresponding temperatures of 40, 60, 80, and 100 °C, respectively (Fig. S17). The increased specific capacitance observed with the SORC separator can be attributed to enhanced charge mobility in the electrolyte under high-temperature conditions. Importantly, the capacitance retention of the SC with SORC separator was found to improve by 1.60 times when comparing 100–25 °C. These findings suggested that the SC with SORC separator possessed stable and superior electrochemical performance over a wide temperature range, making it particularly promising for energy storage applications in high-temperature environments. To demonstrate the potential of SCs using the novel cellulose separator with unique nano-cracks in practical applications, a 3.0 V light-emitting diode (LED) lamp was successfully turned on using three SCs in series (Fig. 6i).

## Conclusions

In summary, we have employed an efficient SIL/DMSO solvent) to fabricate a high-performance regenerated cellulose (SORC) film as the supercapacitor separator. Analysis of the dissolution and regeneration processes of cellulose via PLM images, *in situ* ATR-FTIR, and DFT calculations demonstrated the effectiveness of DMSO in regulating the multiple hydrogen bonding network of cellulose. The presence of DMSO expedited the reassembly of cellulose molecules, resulting in the fabrication of regenerated cellulose separator with unique nano-crack structures. The separator exhibited competitive advantages, including exceptional flexibility, robust mechanical strength (70 MPa), adequate thermal stability, high porosity (70.2%), substantial electrolyte retention (329%), and superior ionic conductivity (13.4 mS cm^−1^). The electrochemical performance of a supercapacitor comprising of KOH electrolyte, SORC separator, and activated carbon electrode was exceptional, with a high specific capacitance of 93.6 F g^−1^ and excellent cyclability (99.5% retention after 10,000 cycles at 1.0 A g^−1^), which significantly outperformed commercialized NKK-MPF30AC and NKK-TF4030. To the best of our knowledge, such a cellulose-based separator with both simple fabrication and outstanding properties has not previously been achieved by established methods. This work presents a sustainable, eco-friendly, and effective strategy for developing flexible and renewable cellulose-based separators with remarkable properties for energy storage devices.

## Supplementary Information

Below is the link to the electronic supplementary material.

Supplementary file1 (DOCX 35016 KB)

Supplementary file2 (MP4 33661 KB)

Supplementary file3 (MP4 18465 KB)

Supplementary file4 (MP4 28854 KB)

Supplementary file5 (MP4 27210 KB)

## References

[1] C. Chen, Y. Zhang, Y. Li, J. Dai, J. Song et al., All-wood, low tortuosity, aqueous, biodegradable supercapacitors with ultra-high capacitance. Energy Environ. Sci. **10**(2), 538–545 (2017). 10.1039/C6EE03716J

[2] J. Li, H. Jia, S. Ma, L. Xie, X.X. Wei et al., Separator design for high-performance supercapacitors: requirements, challenges, strategies, and prospects. ACS Energy Lett. **8**(1), 56–78 (2023). 10.1021/acsenergylett.2c01853

[3] Y. Song, X. Liu, D. Ren, H. Liang, L. Wang et al., Simultaneously blocking chemical crosstalk and internal short circuit via gel-stretching derived nanoporous non-shrinkage separator for safe lithium-ion batteries. Adv. Mater. **34**(2), 2106335 (2022). 10.1002/adma.202106335 10.1002/adma.202106335 34617339

[4] H. Wu, J. Mu, Y. Xu, F. Xu, S. Ramaswamy et al., Heat-resistant, robust, and hydrophilic separators based on regenerated cellulose for advanced supercapacitors. Small **19**(1), 2205152 (2023). 10.1002/smll.202205152 10.1002/smll.202205152 36354185

[5] S. Zhong, B. Yuan, Z. Guang, D. Chen, Q. Li et al., Recent progress in thin separators for upgraded lithium ion batteries. Energy Storage Mater. **41**, 805–841 (2021). 10.1016/j.ensm.2021.07.028

[6] D. Guo, L.Q. Mu, F. Lin, G.L. Liu, Mesoporous polyimide thin films as dendrite-suppressing separators for lithium-metal batteries. ACS Nano **18**(1), 155–163 (2023). 10.1021/acsnano.3c04159 38127801 10.1021/acsnano.3c04159

[7] M. Liu, K. Turcheniuk, W. Fu, Y. Yang, M. Liu et al., Scalable, safe, high-rate supercapacitor separators based on the Al_2_O_3_ nanowire polyvinyl butyral nonwoven membranes. Nano Energy **71**, 104627 (2020). 10.1016/j.nanoen.2020.104627

[8] Z. Zou, Y. Wei, Z. Hu, H. Pu, Synthesis of polypropylene nanofiber separators for lithium-ion batteries via nanolayer coextrusion. Chem. Eng. J. **474**, 145724 (2023). 10.1016/j.cej.2023.145724

[9] X. Dai, X. Zhang, J. Wen, C. Wang, X. Ma et al., Research progress on high-temperature resistant polymer separators for lithium-ion batteries. Energy Storage Mater. **51**, 638–659 (2022). 10.1016/j.ensm.2022.07.011

[10] H. Li, D. Wu, J. Wu, L.Y. Dong, Y.J. Zhu et al., Flexible, high-wettability and fire-resistant separators based on hydroxyapatite nanowires for advanced lithium-ion batteries. Adv. Mater. **29**(44), 1703548 (2017). 10.1002/adma.201703548 10.1002/adma.201703548 29044775

[11] D. Zhou, X. Tang, X. Guo, P. Li, D. Shanmukaraj et al., Polyolefin-based janus separator for rechargeable sodium batteries. Angew. Chem. Int. Ed. **59**(38), 16725–16734 (2020). 10.1002/anie.202007008 10.1002/anie.202007008 32524710

[12] H. Tu, M. Zhu, B. Duan, L. Zhang, Recent progress in high-strength and robust regenerated cellulose materials. Adv. Mater. **33**(28), 2000682 (2021). 10.1002/adma.202000682 10.1002/adma.202000682 32686231

[13] A. Noori, M.F. El-Kady, M.S. Rahmanifar, R.B. Kaner, M.F. Mousavi, Towards establishing standard performance metrics for batteries, supercapacitors and beyond. Chem. Soc. Rev. **48**(5), 1272–1341 (2019). 10.1039/c8cs00581h 30741286 10.1039/c8cs00581h

[14] R. Mendoza-Jiménez, J. Oliva, K.P. Padmasree, A.I. Mtz-Enriquez, C.R. Garcia, Enhancement of capacitance of waterproof supercapacitors by controlling the thickness of their composite electrodes (graphene/La_0.2_Gd_1.8_Zr_2_O_7_: La_0.7_Sr_0.3_MnO_3_). Ceram. Int. **50**(12), 21827–21838 (2024). 10.1016/j.ceramint.2024.03.295

[15] R. Mendoza, M. Al-Sardar, A.I. Oliva, G. Robledo-Trujillo, V. Rodriguez-Gonzalez et al., Improving the electrochemical performance of flexible carbon nanotubes based supercapacitors by depositing Ni@TiO_2_: W nanoparticles on their anodes. J. Phys. Chem. Solids **155**, 110128 (2021). 10.1016/j.jpcs.2021.110128

[16] R. Mendoza, M. Balderas-Soto, R.G. Suarez, J. Zamora, A.I. Mtz-Enriquez et al., Role of the MnCoGe alloys to enhance the capacitance of flexible supercapacitors made with electrodes of recycled aluminum and carbon nanotubes. Synth. Met. **306**, 117654 (2024). 10.1016/j.synthmet.2024.117654

[17] H.Z. Chen, Z.C. Wang, Y.T. Feng, S.Y. Cai, H.P. Gao et al., Cellulose-based separators for lithium batteries: source, preparation and performance. Chem. Eng. J. **471**, 144593 (2023). 10.1016/j.cej.2023.144593

[18] H. Wu, H. Huang, Y. Xu, F. Xu, X. Zhang, Ultrathin separator with efficient ion transport and superior stability prepared from cotton cellulose for advanced supercapacitors. Chem. Eng. J. **470**, 144089 (2023). 10.1016/j.cej.2023.144089

[19] L. Yao, K. Zheng, N. Koripally, N. Eedugurala, J.D. Azoulay, X. Zhang, T.N. Ng, Structural pseudocapacitors with reinforced interfaces to increase multifunctional efficiency. Sci. Adv. **9**(25), adh0069 (2023). 10.1126/sciadv.adh0069 10.1126/sciadv.adh0069 PMC10289652 37352340

[20] D. Zhao, C. Chen, Q. Zhang, W. Chen, S. Liu et al., High performance, flexible, solid-state supercapacitors based on a renewable and biodegradable mesoporous cellulose membrane. Adv. Energy Mater. **7**(18), 1700739 (2017). 10.1002/aenm.201700739

[21] R. Mendoza, J. Oliva, V. Rodriguez-Gonzalez, Effect of the micro-, meso- and macropores on the electrochemical performance of supercapacitors: a review. Int. J. Energy Res. **46**(6), 6989–7020 (2022). 10.1002/er.7670

[22] B. Dyatkin, V. Presser, M. Heon, M.R. Lukatskaya, M. Beidaghi et al., Development of a green supercapacitor composed entirely of environmentally friendly materials. ChemSusChem **6**(12), 2269–2280 (2013). 10.1002/cssc.201300852 24136900 10.1002/cssc.201300852

[23] S. Wang, A. Lu, L. Zhang, Recent advances in regenerated cellulose materials. Prog. Polym. Sci. **53**, 169–206 (2016). 10.1016/j.progpolymsci.2015.07.003

[24] L. Zhang, W. Shi, H. Sheng, S. Feng, M. Yao et al., Unique CO_2_-switched cellulose solution properties in the CO_2_/DBU/DMSO solvent system and the preparation of regenerated materials. Green Chem. **23**(16), 5856–5865 (2021). 10.1039/D1GC01771C

[25] B. Medronho, B. Lindman, Brief overview on cellulose dissolution/regeneration interactions and mechanisms. Adv. Colloid Interface Sci. **222**, 502–508 (2015). 10.1016/j.cis.2014.05.004 24931119 10.1016/j.cis.2014.05.004

[26] X. Li, H. Li, Z. Ling, D. Xu, T.T. You et al., Room-temperature superbase-derived ionic liquids with facile synthesis and low viscosity: powerful solvents for cellulose dissolution by destroying the cellulose aggregate structure. Macromolecules **53**(9), 3284–3295 (2020). 10.1021/acs.macromol.0c00592

[27] R.P. Swatloski, S.K. Spear, J.D. Holbrey, R.D. Rogers, Dissolution of cellose with ionic liquids. J. Am. Chem. Soc. **124**(18), 4974–4975 (2002). 10.1021/ja025790m 11982358 10.1021/ja025790m

[28] H. Wang, G. Gurau, R.D. Rogers, Ionic liquid processing of cellulose. Chem. Soc. Rev. **41**(4), 1519–1537 (2012). 10.1039/C2CS15311D 22266483 10.1039/c2cs15311d

[29] A. Pinkert, K.N. Marsh, S. Pang, M.P. Staiger, Ionic liquids and their interaction with cellulose. Chem. Rev. **109**(12), 6712–6728 (2009). 10.1021/cr9001947 19757807 10.1021/cr9001947

[30] Xu. Daman, G. Teng, Y. Heng, Z. Chen, Hu. Dongying, Eco-friendly and thermally stable cellulose film prepared by phase inversion as supercapacitor separator. Mater. Chem. Phys. **249**, 122979 (2020). 10.1016/j.matchemphys.2020.122979

[31] W. Liu, K. Liu, H. Du, T. Zheng, N. Zhang et al., Cellulose nanopaper: fabrication, functionalization, and applications. Nano-Micro Lett. **14**(1), 104 (2022). 10.1007/s40820-022-00849-x 10.1007/s40820-022-00849-x PMC9008119 35416525

[32] L. Szabó, R. Milotskyi, G. Sharma, K. Takahashi, Cellulose processing in ionic liquids from a materials science perspective: turning a versatile biopolymer into the cornerstone of our sustainable future. Green Chem. **25**(14), 5338–5389 (2023). 10.1039/d2gc04730f

[33] S. Livazovic, Z. Li, A.R. Behzad, K.V. Peinemann, S.P. Nunes, Cellulose multilayer membranes manufacture with ionic liquid. J. Membr. Sci. **490**, 282–293 (2015). 10.1016/j.memsci.2015.05.009

[34] M.E. Lamm, K. Li, J. Qian, L. Wang, N. Lavoine, R. Newman et al., Recent advances in functional materials through cellulose nanofiber templating. Adv. Mater. **33**(12), 2005538 (2021). 10.1002/adma.202005538 10.1002/adma.202005538 33565173

[35] E. Lizundia, D. Kundu, Advances in natural biopolymer-based electrolytes and separators for battery applications. Adv. Funct. Mater. **31**(3), 2005646 (2021). 10.1002/adfm.202005646

[36] Y. Xie, H. Zhu, R. Zeng, B. Na, S. Zou et al., Chemical foaming integrated polydopamine hybridization towards high-performance cellulose-based separators for ultrastable and high-rate lithium metal batteries. J. Power Sources **538**, 231562 (2022). 10.1016/j.jpowsour.2022.231562

[37] A.D. Becke, Density-functional thermochemistry. I. The effect of the exchange-only gradient correction. J. Chem. Phys. **96**(3), 2155–2160 (1992). 10.1063/1.462066

[38] P.J. Stephens, F.J. Devlin, C.F. Chabalowski, M.J. Frisch, Ab initio calculation of vibrational absorption and circular dichroism spectra using density functional force fields. J. Phys. Chem. **98**(45), 11623–11627 (1994). 10.1021/j100096a001

[39] F. Weigend, R. Ahlrichs, Balanced basis sets of split valence, triple zeta valence and quadruple zeta valence quality for H to Rn: design and assessment of accuracy. Phys. Chem. Chem. Phys. **7**(18), 3297–3305 (2005). 10.1039/B508541A 16240044 10.1039/b508541a

[40] S. Grimme, J. Antony, S. Ehrlich, H. Krieg, A consistent and accurate ab initio parametrization of density functional dispersion correction (DFT-D) for the 94 elements H-Pu. J. Chem. Phys. **132**(15), 154104 (2010). 10.1063/1.3382344 20423165 10.1063/1.3382344

[41] A.V. Marenich, C.J. Cramer, D.G. Truhlar, Universal solvation model based on solute electron density and on a continuum model of the solvent defined by the bulk dielectric constant and atomic surface tensions. J. Phys. Chem. B **113**(18), 6378–6396 (2009). 10.1021/jp810292n 19366259 10.1021/jp810292n

[42] T. Lu, F. Chen, Multiwfn: a multifunctional wavefunction analyzer. J. Comput. Chem. **33**(5), 580–592 (2012). 10.1002/jcc.22885 22162017 10.1002/jcc.22885

[43] W. Humphrey, A. Dalke, K. Schulten, VMD: visual molecular dynamics. J. Molec. Graphics. **14**(1), 33–38 (1996). 10.1016/0263-7855(96)00018-5 10.1016/0263-7855(96)00018-5 8744570

[44] Y. Zhou, X.C. Zhang, D.X. Yin, J.M. Zhang, Q.Y. Mi et al., The solution state and dissolution process of cellulose in ionic-liquid-based solvents with different hydrogen-bonding basicity and microstructures. Green Chem. **24**(9), 3824–3833 (2022). 10.1039/d2gc00374k

[45] X.Y. Wang, T.T. You, W.Q. Zheng, X. Li, S. Chen et al., Efficient fabrication of cellulose nanofibers with novel superbase-derived ionic liquid/co-solvents: rapid cellulose dissolution and improved solution electrospinnability. Chem. Eng. J. **483**, 148841 (2024). 10.1016/j.cej.2024.148841

[46] H.L. Li, M. Kruteva, M. Dulle, Z. Wang, K. Mystek et al., Understanding the drying behavior of regenerated cellulose gel beads: the effects of concentration and nonsolvents. ACS Nano **16**(2), 2608–2620 (2022). 10.1021/acsnano.1c09338 35104108 10.1021/acsnano.1c09338 PMC8867908

[47] B.S. Beckingham, N.A. Lynd, D.J. Miller, Monitoring multicomponent transport using *in situ* ATR FTIR spectroscopy. J. Membr. Sci. **550**, 348–356 (2018). 10.1016/j.memsci.2017.12.072

[48] B.R. Liu, W.H. Li, Y. Xu, H. Zhang, R.W.M. Cai et al., Mechanism of cellulose regeneration from its ionic liquid solution as revealed by infrared spectroscopy. Polymer **257**, 125280 (2022). 10.1016/j.polymer.2022.125280

[49] N. Dissanayake, V.D. Thalangamaarachchige, M. Thakurathi, M. Knight, E.L. Quitevis et al., Dissolution of cotton cellulose in 1:1 mixtures of 1-butyl-3-methylimidazolium methylphosphonate and 1-alkylimidazole co-solvents. Carbohydr. Polym. **221**, 63–72 (2019). 10.1016/j.carbpol.2019.05.071 31227168 10.1016/j.carbpol.2019.05.071

[50] Q.Z. Li, G.S. Wu, Z.W. Yu, The role of methyl groups in the formation of hydrogen bond in dmso-methanol mixtures. J. Am. Chem. Soc. **128**(5), 1438–1439 (2006). 10.1021/ja0569149 16448100 10.1021/ja0569149

[51] H.P. Fink, P. Weigel, H.J. Purz, J. Ganster, Structure formation of regenerated cellulose materials from nmmo-solutions. Prog. Polym. Sci. **26**(9), 1473–1524 (2001). 10.1016/S0079-6700(01)00025-9

[52] S. Dixit, J. Crain, W.C.K. Poon, J.L. Finney, A.K. Soper, Molecular segregation observed in a concentrated alcohol–water solution. Nature **416**(6883), 829–832 (2002). 10.1038/416829a 11976678 10.1038/416829a

[53] T. Lu, Q.X. Chen, Independent gradient model based on hirshfeld partition: a new method for visual study of interactions in chemical systems. J. Comput. Chem. **43**(8), 539–555 (2022). 10.1002/jcc.26812 35108407 10.1002/jcc.26812

[54] P. Heasman, A.Y. Mehandzhiyski, S. Ghosh, I. Zozoulenko, A computational study of cellulose regeneration: all-atom molecular dynamics simulations. Carbohydr. Polym. **311**, 120768 (2023). 10.1016/j.carbpol.2023.120768 37028861 10.1016/j.carbpol.2023.120768

[55] B.T. Yuan, K.C. Wen, D.J. Chen, Y.P. Liu, Y.F. Dong et al., Composite separators for robust high rate lithium ion batteries. Adv. Funct. Mater. **31**(32), 2101420 (2021). 10.1002/adfm.202101420

[56] P. Zugenmaier, Conformation and packing of various crystalline cellulose fibers. Prog. Polym. Sci. **26**(9), 1341–1417 (2001). 10.1016/S0079-6700(01)00019-3

[57] L. Geng, X. Peng, C. Zhan, A. Naderi, P.R. Sharma et al., Structure characterization of cellulose nanofiber hydrogel as functions of concentration and ionic strength. Cellulose **24**(12), 5417–5429 (2017). 10.1007/s10570-017-1496-2

[58] X.F. Wang, X.H. Lu, B. Liu, D. Chen, Y.X. Tong et al., Flexible energy-storage devices: design consideration and recent progress. Adv. Mater. **26**(28), 4763–4782 (2014). 10.1002/adma.201400910 24913891 10.1002/adma.201400910

[59] H. Yang, X. Shi, S. Chu, Z. Shao, Y. Wang, Design of block-copolymer nanoporous membranes for robust and safer lithium-ion battery separators. Adv. Sci. **8**(7), 2003096 (2021). 10.1002/advs.202003096 10.1002/advs.202003096 PMC8025019 33854886

[60] W. Zhou, M. Yang, M. Chen, G. Zhang, X. Han et al., Ion-sieving effect enabled by sulfonation of cellulose separator realizing dendrite-free Zn deposition. Adv. Funct. Mater. **34**(27), 2315444 (2024). 10.1002/adfm.202315444

[61] H. Ma, J. Yu, M. Chen, X. Han, J. Chen et al., Amino-enabled desolvation sieving effect realizes dendrite-inhibiting thin separator for durable aqueous zinc-ion batteries. Adv. Funct. Mater. **33**(52), 2307384 (2023). 10.1002/adfm.202307384

[62] Y. Yang, W. Wang, G. Meng, J. Zhang, Function-directed design of battery separators based on microporous polyolefin membranes. J. Mater. Chem. A **10**(27), 14137–14170 (2022). 10.1039/D2TA03511A

[63] L.H. Yu, J.S. Miao, Y. Jin, J.Y.S. Lin, A comparative study on polypropylene separators coated with different inorganic materials for lithium-ion batteries. Front. Chem. Sci. Eng. **11**(3), 346–352 (2017). 10.1007/s11705-017-1648-9

[64] M.F. Lagadec, R. Zahn, V. Wood, Characterization and performance evaluation of lithium-ion battery separators. Nat. Energy **4**(1), 16–25 (2019). 10.1038/s41560-018-0295-9

[65] Z. Tang, S. Li, Y. Li, H. Xu, Y. Yu et al., Lithium metal electrode protected by stiff and tough self-compacting separator. Nano Energy **69**, 104399 (2020). 10.1016/j.nanoen.2019.104399

[66] Q. Zhang, C. Chen, W. Chen, G. Pastel, X. Guo et al., Nanocellulose-enabled, all-nanofiber, high-performance supercapacitor. ACS Appl. Mater. Interfaces **11**(6), 5919–5927 (2019). 10.1021/acsami.8b17414 30657318 10.1021/acsami.8b17414

