# Supplementary material for: Robust and High-Wettability Cellulose Separators with Molecule-Reassembled Nano-Cracked Structures for High-Performance Supercapacitors
Source: Nanomicro Lett. 2025 Feb 19;17:153. doi: 10.1007/s40820-025-01650-2 (PMC11839970; doi:10.1007/s40820-025-01650-2)
Supplement: Supplementary file 1 — Supplementary file1 (DOCX 35016 KB) [file 40820_2025_1650_MOESM1_ESM.docx]

Supporting Information for

**Robust and High-Wettability Cellulose Separators with Molecule-Reassembled Nano-Cracked Structures for High-Performance Supercapacitors**

Xiaoyu Wang^1^, Wenqiu Zheng^1^, Hui Zhao^1^, Junying Li^1^, Sheng Chen^1,^ *, and Feng Xu^1,^ *

^1^ State Key Laboratory of Efficient Production of Forest Resources, Beijing Key Laboratory of Lignocellulosic Chemistry, Beijing Forestry University, Beijing 100083, P. R. China

*Corresponding authors. E-mail: [shengchen@bjfu.edu.cn](mailto:shengchen@bjfu.edu.cn) (Sheng Chen); [xfx315@bjfu.edu.cn](mailto:xfx315@bjfu.edu.cn) (Feng Xu)

**S1 Experimental Section**

**S1.1 Characterization Methods**

***S1.1.1 Characterization of Physical Properties for Solvents and Cellulose Solutions***

The viscosity (*η*) measurements of solvents were performed with a rolling ball viscosimeter (Lovis 2000 M, Anton Paar). The electrical conductivities of solvents were measured by a conductivity meter with a built-in temperature sensor (320C-01A, JS Instruments, Shanghai). The dissolution process of pulp fibers was captured by a polarized light microscopy (PLM) (Olympus Corporation Tokyo 163-0914) equipped with a transparent thermostatic hot stage (HS101, Cossim). Samples were pressed with glass slides and put on the heating device to record the dissolution process. The rheological behaviors of cellulose solutions were measured by a rotational rheometer (MCR 92, Anton Paar, Austria) equipped with a parallel plate device (50 mm plate diameter, 1 mm gap size). Viscosity measurements were performed from 25 to 100 °C at a heating rate of 5 °C·min^-1^ with a fixed shear rate of 10 s^-1^. Steady shear measurements and dynamic oscillatory shear experiments were performed with a shear rate ranging from 0.1 to 1000 s^-1^ and angular frequency ranging from 0.1 to 100 rad·s^-1^, respectively. A strain amplitude of 10% was chosen to fall well into the linear viscoelastic regime for the measurement in oscillation mode.

***S1.1.2 In-situ Infrared Characterization for Regeneration Process of Cellulose***

Fourier transform infrared (FTIR) spectroscopy was performed on a Bruker tensor 70 spectrometer equipped with an (Attenuated total reflection) ATR detector. FTIR spectra were collected at 4 cm^-1^ resolution with 16 scans in the wavenumber range from 4000 to 650 cm^-1^. FTIR spectra during the regeneration process of cellulose at ambient temperature (25 °C) were recorded continuously at 5 min interval. All FTIR spectra were baseline corrected and smoothed.

***S1.1.3 Characterization of Structure and Properties for Separators***

The surface morphology was assessed by Scanning electron microscopy (SEM) (SU8010, Japan) under a high vacuum condition at acceleration voltages of 3.0 kV. Separators were coated with gold using a vacuum sputter coater before observation. The wide-angle X-ray scattering (WAXS) measurements were carried out on a PANalytical Empyrean diffractometer with the Cu(K_α_) radiation source (λ=1.5406Å). The diffractometer was operated at 40 kV and 40 mA at the scanning of 2^o^·min^-1^ in the range of 2θ=5-50^o^. Small-angle X-ray scattering (SAXS) measurements were performed on a small-angle scatterometer (Nano-inXider, Xenocs) with the Cu(Kα) radiation source (λ=1.5406Å). The sample-to-detector distance of 1.185 m was used to allow a scattering vector (*q*) range of 0.07-0.25 Å^-1^. The two-dimensional scattering (2D-SAXS) patterns were collected using a Pilatus 3R 300K camera (pixel size, 172×172 μm). The water or electrolyte contact angles of separators were measured using a contact angle meter (SL200KS, KINO). The thermal property was characterized by a TGA/SDTA851e (Mettler Toledo) instrument. Separators were heated in the range of 30-600 ^o^C with a ramping ratio of 10 ^o^C/min under a nitrogen atmosphere. The transmittance was measured on a UV-vis spectrophotometer (UV-2600). Spectra were recorded over the range of 200-800 nm with a resolution of 1 nm. The tensile stress was measured using a Zwick/Roell instrument. All the tensile tests were measured by referring to the standard of ASTM D882. The porosity of separators was measured using the n-butanol uptake method. The mass of separators was recorded before and after soaking with n-butanol for 6 h. The excess of n-butanol on the wet separators was wiped off with tissue paper before the determination of the separator mass. The porosity was calculated using Eq. S1:

$P\left( \% \right)=\frac{{M_{b}}/{\rho_{b}}}{{M_{p}}/{\rho_{p}+{M_{b}}/{\rho_{b}}}}\times100\%$ (S1)

where *P* is the porosity of the separator (%), *M_p_* is the mass of the separator (g), *M_b_* is the mass of absorbed n-butanol (g), *ρ_p_* is the density of the separator (g·cm^-3^), and *ρ_b_* is the density of n-butanol (0.81 g·cm^-3^).

Electrolyte uptake of separators was obtained by measuring the weight of the separator before (*W_0_*, g) and after (*W_1_*, g) soaking in the liquid electrolyte (6 M KOH) for 48 h. The surface of the separator was then wiped with tissue paper to remove excess electrolyte before weighing. The KOH uptake (wt%) was calculated using Eq. S2:

$KOH uptake \left( wt\% \right)=\frac{W_{1}-W_{0}}{W_{0}}\times100\%$ (S2)

The ionic conductivity (σ, S/cm) was calculated using Eq. S3:

$\sigma=\frac{L}{R_{b}\times A}$ (S3)

where *L* (cm) is the distance between the two electrodes, *A* (cm^2^) is the effective area of the separator, and *R_b_* (Ω) is the bulk resistance of the separator/KOH polymer electrolyte.

***S1.1.4 Characterization of Electrochemical Performance for Supercapacitors***

The electrochemical performance of supercapacitors with either RC films or commercial separators were evaluated by cyclic voltammetry (CV), electrochemical impedance spectroscopy (EIS), and galvanostatic charge-discharge (GCD) using an AUT87226 electrochemical workstation (Metrohm Autolab B.V.). The electrochemical characterizations were carried out under ambient temperature with 6 M KOH as an electrolyte. The CV experiment was conducted in a potential window of 0-1.0 V at various scan rates from 10 to 100 mV·s^-1^. The impendence behavior of the supercapacitor was analyzed by the EIS in the frequency region of 0.01-100 kHz. GCD measurements were also performed within a potential window of 0-1.0 V at current densities of 0.25-10.0 A·g^-1^. The specific capacitance (F·g^-1^) of the supercapacitor was estimated from the GCD profiles according to Eq. S4:

$C_{s}=\frac{4\times I\times\Delta t}{m\times\Delta V}$ (S4)

where *I* (A) is the discharge current, *m* (g) is the total mass of active materials in the two electrodes, *ΔV* (V) is the voltage window on discharge (excluding the voltage drop), and *Δt* (s) is the time duration for a full discharge.

The energy density (*E*, Wh·kg^-1^) and power density (*P*, W·kg^-1^) of the supercapacitor were estimated using Eqs. S5 and S6:

$E=\frac{C_{s}\times{\Delta V}^{2}}{3.6\times2}$ (S5)

$P=\frac{E\times3600}{\Delta t}$ (S6)

where *Cs* (F·g^-1^) is the specific capacitance of the supercapacitor, *ΔV* (V) is the voltage window on discharge (excluding the voltage drop), and *Δt* (s) is the time duration for a full discharge.

**S2 Results and Discussion**

**S2.1 Optimization of DMSO Content**

According to previous studies, the combination of co-solvents such as DMSO, *N, N*-dimethylacetamide (DMAc), and *N, N*-dimethylformamide (DMF) with ILs contributed to cellulose dissolution [S1, S2]. However, both small and excessive amounts of co-solvent are unfavorable for the solubility and dissolution efficiency of cellulose. In this study, the viscosity of SIL/DMSO solvents (e.g., 1.680 Pa·s at a mass ratio of 5:5) substantially decreased as compared with pure SIL (0.022 Pa·s), which is beneficial for the preparation and subsequent processing of cellulose solutions (Fig. S1a). Meanwhile, SIL/DMSO solvents exhibited high ionic conductivity (e.g., 1.43 mS·cm^-1^ at a mass ratio of 5:5), indicating their significant potential as electrolytes in electrochemistry applications (Fig. S1b) [S3]. Moreover, the dissolution time of 4 wt% cellulose in SIL/DMSO solvents showed a decreasing trend initially and then increased with increasing DMSO content, reaching a minimum at a SIL to DMSO mass ratio of 7:3 (Fig. S1c). The dissolution time of 4 wt% cellulose in SIL/DMSO (7:3) (5 min) was reduced by 89.4% compared to that in pure SIL (47 min). Hence, the optimal mass ratio of SIL to DMSO was determined as 7:3, which significantly enhanced the efficiency of cellulose dissolution and would be beneficial to the scale-up fabrication of cellulose-based separators.


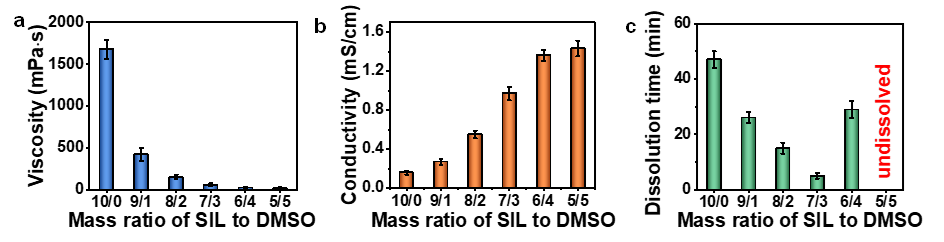


**Fig. S1** Optimization of DMSO content. **a** Viscosity and **b** conductivity of SIL/DMSO solvent systems with various mass ratio of SIL to DMSO. **c** Dissolution time of 4 wt% DWP in SIL/DMSO solvent systems with various mass ratio of SIL to DMSO

**S2.2** **Hydrogen Bonds and Binding Energy Between the Components in Complexes**

To further investigate the interaction mechanism between SIL and cellulose and to clarify the role of DMSO in cellulose dissolution, DFT calculations were performed to analyze the hydrogen bonds and interaction energies between cellulose (with cellobiose as a model compound), DMSO, and the SIL anion and cation (Fig. S2). In the SIL-DMSO-cellobiose, multiple hydrogen bonding interactions were formed between the components. As exhibited in Table S1, the binding energy between components followed as: cellobiose-SIL (-13.81 kcal/mol) > DMSO-SIL cation (-5.72 kcal/mol) > SIL cation-SIL anion (-3.95 kcal/mol). This result suggested there is a strong hydrogen bonding interaction between DMSO and SIL cations, and that DMSO does not significantly affect the hydrogen bonding interaction between cellulose and SIL.


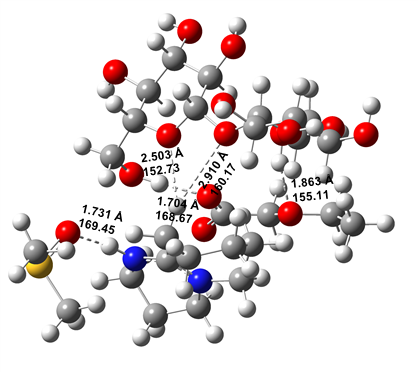


**Fig. S2** The optimized structure of SIL-DMSO-cellobiose

**Table S1** Binding energy of hydrogen bonds between the components in complexes

| **Complex** | **Component-component** | **Binding energy (kcal/mol)** |
| --- | --- | --- |
| SIL | SIL_Ion+_-SIL_Ion-_ | -8.57 |
| SIL-DMSO | SIL_Ion_-DMSO | -5.82 |
|  | SIL_Ion+_-DMSO | -5.11 |
|  | SIL_Ion-_-DMSO | 0.13 |
|  | SIL_Ion+_-SIL_Ion-_ | -4.96 |
| SIL-DMSO-cellobiose | SIL_Ion_-DMSO-Cellobiose | -20.43 |
|  | SIL_Ion_-Cellobiose | -13.81 |
|  | SIL_Ion_-DMSO | -6.59 |
|  | DMSO-Cellobiose | 5.77 |
|  | SIL_Ion+_-DMSO | -5.72 |
|  | SIL_Ion+_-SIL_Ion-_ | -3.95 |

**S2.3 Dissolution Mechanism of Cellulose in the SIL/DMSO Solvent**

Cellulose, a crystalline biopolymer composed of repeated glucose units linked by *β*-1,4-glycosidic bonds, has a robust intra-/inter-molecular hydrogen-bonding network [S4]. The effective dissolution of cellulose in pure SIL was mainly attributed to the protonated cations and active anions [S5]. The addition of DMSO to SIL significantly decreased the solvent viscosity, thereby expediting the transportation of SIL ions to cellulose chains. Moreover, DMSO acted as a hydrogen bonding acceptor interacting with SIL cations through the hydrogen bonds, forming small ion clusters (Fig. S3) [S6]. Meanwhile, the solvated cations of SIL lead to more free anions. These small ion clusters and free anions may permeate the hierarchical structures of cellulose, significantly augmenting the dissolution efficiency.

**Fig. S3** Schematic of potential interactions of hydrogen bonds before and after cellulose dissolution

**S2.4 Rheology analysis of cellulose/SIL/DMSO solutions**

A homogeneous cellulose solution with excellent fluidity was obtained by the efficient dissolution of cellulose in SIL/DMSO solvent (Fig. S4a). To gain a better insight into the practical processing of the cellulose/SIL/DMSO solution, the steady shear and dynamic viscoelastic properties were investigated (Fig. S4b-f). Specifically, Fig. S4b revealed a substantial decrease in the viscosity of cellulose solutions with an increase in temperature from 25 to 100 °C. The viscosity values of the cellulose/SIL/DMSO solution were consistently lower than those of the cellulose/SIL solution. Additionally, the cellulose/SIL/DMSO solution exhibited typical shear-thinning behavior at 25 °C, in which the gradual untangling and orientation of cellulose chains as the shear rate increased (Fig. S4c) [S7]. Compared with the cellulose/SIL solution that showed dominant elastic behavior, the cellulose/SIL/DMSO solution displayed the characteristic of a viscous-like liquid (*G′* < *G′′*; tanδ > 1) within the tested angular frequency range, demonstrating its excellent spreadability and processability (Fig. S4d, e) [S8]. Notably, the Cox−Merz rule (an empirical rule states $\eta^{*}\left( \omega\right)\cong\eta(\dot{\gamma})|_{\omega=\dot{\gamma}}$) was valid for the cellulose/SIL/DMSO solution only in Newtonian region, whereas slightly higher values of shear viscosities in the shear thinning regime were observed as compared to complex viscosities, suggesting a structural transition of cellulose in this regime (Fig. S4f) [S9].


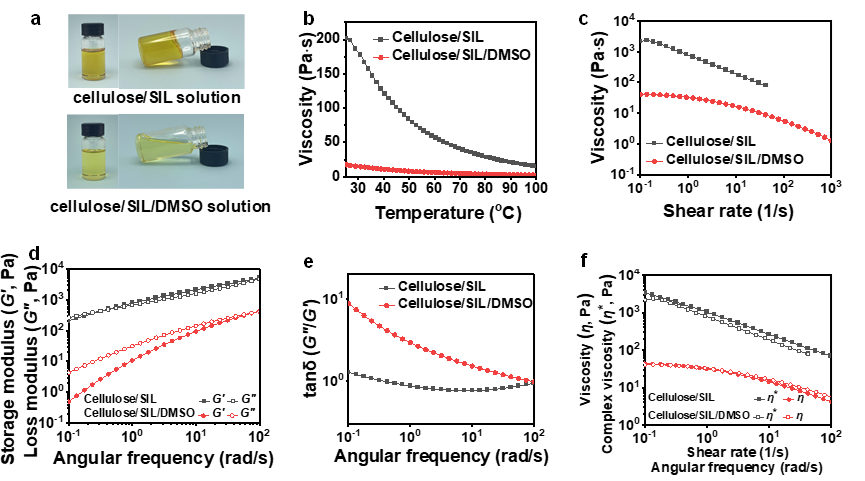


**Fig. S4** Rheology analysis of cellulose/SIL/DMSO solutions. **a** Optical photographs of cellulose solutions. Viscosity curves of cellulose solutions as a function of **b** temperature and **c** shear rate. Frequency dependence of **d** moduli and **e** tan*δ* (ratio of *G″* to *G′*) of cellulose solutions at room temperature. **f** Cox-Merz plot of cellulose solutions at room temperature


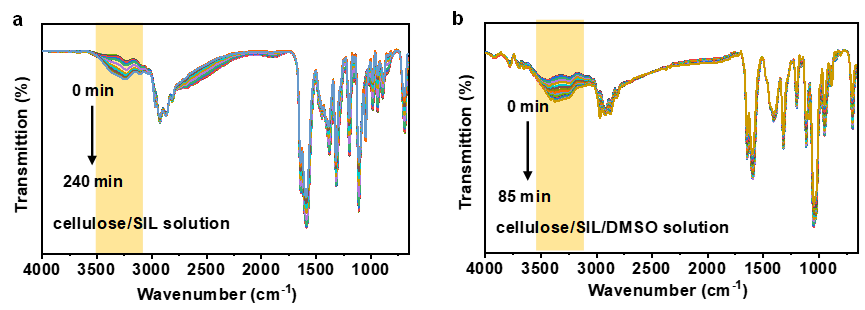


**Fig. S5** Time-dependent FTIR spectra of in situ regeneration from **a** cellulose/SIL and **b** cellulose/SIL/DMSO solutions

**Table S2** Binding energies between the components in complexes

| **Complex** | **Component-component** | **Binding energy (kcal/mol)** |
| --- | --- | --- |
| Ethanol-SIL | EtOH-Ion | -19.55 |
|  | EtOH-Ion- | -11.27 |
|  | EtOH-Ion+ | -3.94 |
|  | Ion+-Ion- | -4.65 |
| Ethanol-DMSO | EtOH-DMSO | -7.72 |
| Ethanol-cellobiose | EtOH-Cell | -16.23 |


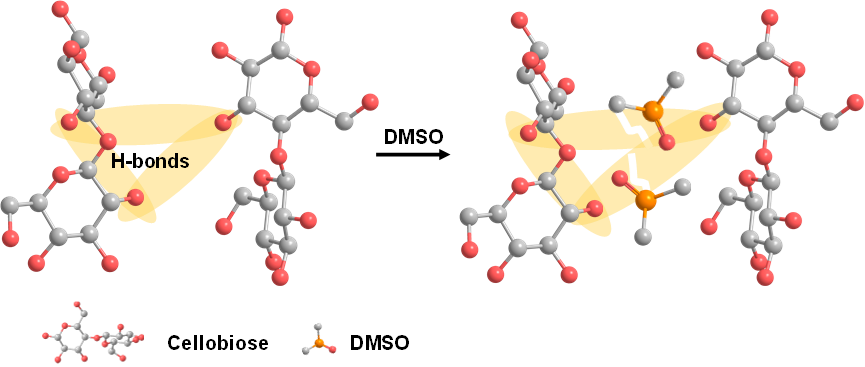


**Fig. S6** Schematic of DMSO in inhibiting the formation of intermolecular hydrogen bonds during cellulose regeneration process.

The inhibition effect occurred through two pathways. Firstly, DMSO molecules formed hydrogen bonds with ethanol, effectively dispersing and reducing the formation of hydrogen bonds between cellulose and ethanol. DMSO also hampered the formation of hydrogen bonds between hydroxyl groups of cellulose through its steric effect. Secondly, the amphiphilic nature of cellulose is determined by the orientation of cellulose chains [S10]. The equatorial orientation of hydroxyl groups and the axial orientation of C-H bonds contribute to the hydrophilicity and hydrophobicity of the glucopyranose ring, respectively. The hydrophobic -CH3 moieties in DMSO promoted interactions between cellulose chains in an axial plane, inhibiting the corresponding formation of intermolecular hydrogen bonds in the equatorial plane of the glucopyranose ring.

**Fig. S7** Optical photographs of SRC and SORC films

**Fig. S8** Surface SEM images of (**a**) SRC and (**b**) SORC films. The insert images represent the water contact angles of RC films


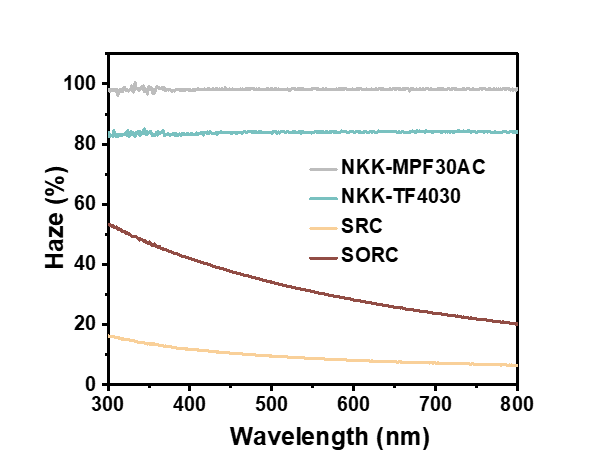


**Fig. S9** Haze curves of NKK-MPF30AC, NKK-TF4030, SRC, and SORC separators

**Fig. S10** Contact angle of the SORC separator after dropping a KOH electrolyte for different times at 1 s, 60 s, 120 s, and 180 s





**Fig. S11** **a** Assembling SC in a CR2432-type coin cell. **b** Schematic diagram of charge/discharge process of SCs

**Table S3** Specific capacitances for previous supercapacitors reported in the literature, which were made with cellulose-based separators

| Separator composition | Electrode | Electrolyte | Capacitance | Capacitance retention | Refs. |
| --- | --- | --- | --- | --- | --- |
| Mesoporous cellulose membrane | Activated carbon | 6 M KOH | 110 F/g at 1.0 A/g | 84.7%, 10,000 cycles | [S11] |
| Porous regenerated cellulose film | Activated carbon | 6 M KOH | 62.5 F/g at 0.5 A/g | 97%, 10,000 cycles | [S12] |
| CNF membrane | Nonocellulose carbon | 6 M KOH | 64.83 F/g at 0.25 A/g | 93%, 1000 cycles | [S13] |
| CNF membrane | CNF/reduced graphene oxide/polypyrrole | 1 M H_2_SO_4_ | 625.6 F/g at 0.22 A/g | 75.4%, 5000 cycles | [S14] |
| Acetate cellulose ester membrane | Activated carbon | 1 M CH_3_COONa | 89.6 F/g | - | [S15] |
| Commercial cellulose membrane (NKK-TF4030) | Activated carbon | 6 M KOH | 72 F/g at 1.0 A/g | 97%, 10,000 cycles | This work |
| Nano-cracked cellulose film | Activated carbon | 6 M KOH | 93.6 F/g at 1.0 A/g | 99.5%, 10,000 cycles | This work |

**Fig. S12** Coulombic efficiency of the SC with a SORC separator

**Fig. S13** Ragone plots of SCs with different separators

**
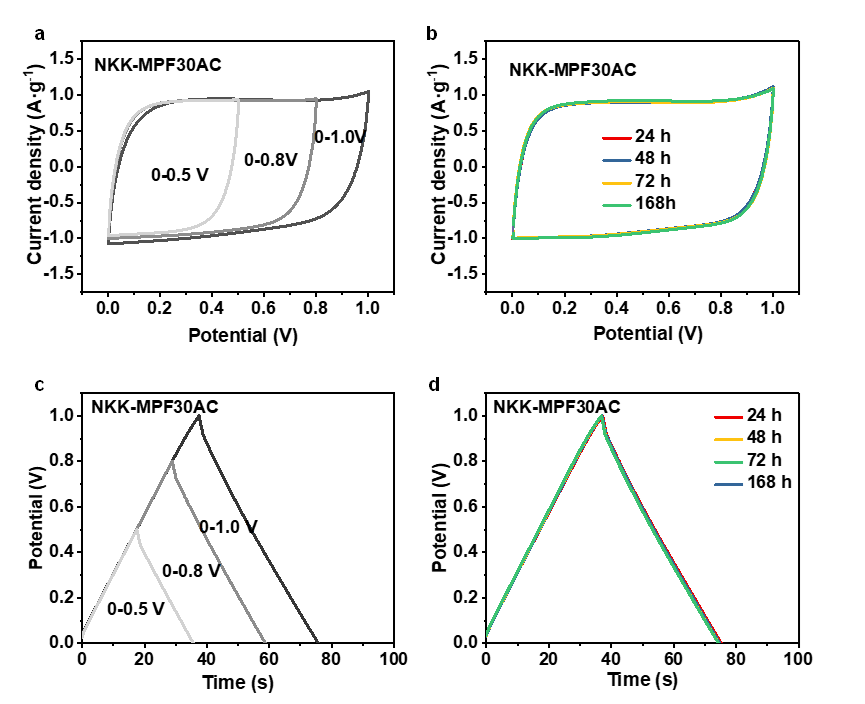
**

**Fig. S14** Lifetime stability of SCs using NKK-MPF30AC separators. **a**, **b** CV curves at a scan rate of 50 mV/s. **c**, **d** GCD profiles at a current density of 1.0 A/g

**
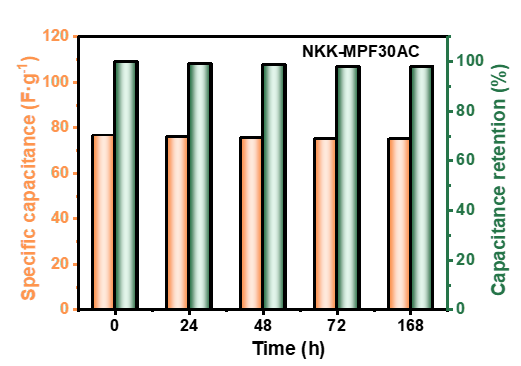
**

**Fig. S15** Specific capacitances and capacitance retention of the SC using NKK-MPF30AC as the separator, measured at different time internals

**
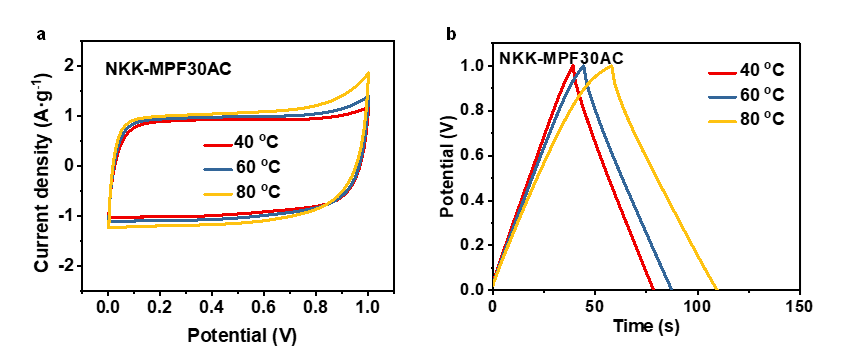
**

**Fig. S16** Heat resistance of SCs using NKK-MPF30AC separators. **a** CV curves at a scan rate of 50 mV/s. **b** GCD profiles at a current density of 1.0 A/g

**
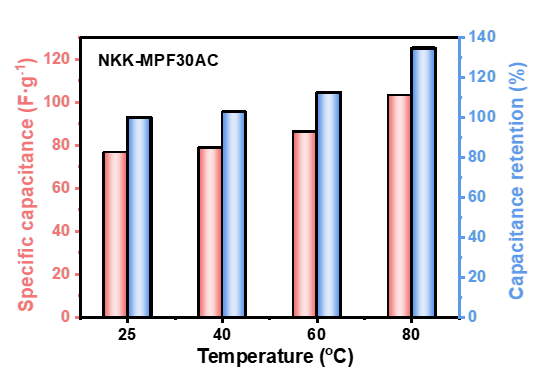
**

**Fig. S17** Specific capacitances and capacitance retention of SCs using NKK-MPF30AC separators, measured at different temperatures

**Supplementary References**

1. Y. Zhou, X. C. Zhang, D. X. Yin, J. M. Zhang, Q. Y. Mi et al., The solution state and dissolution process of cellulose in ionic-liquid-based solvents with different hydrogen-bonding basicity and microstructures. Green Chem. **24**(9), 3824-3833 (2022). <https://doi.org/10.1039/d2gc00374k>
2. L. Zhang, C. Huang, C. Zhang, H. Pan. Swelling and dissolution of cellulose in binary systems of three ionic liquids and three co-solvents. Cellulose **28**(8), 4643-4653 (2021). <https://doi.org/10.1007/s10570-021-03844-4>
3. D. R. MacFarlane, M. Forsyth, P. C. Howlett, J. M. Pringle, J. Sun et al., Ionic liquids in electrochemical devices and processes: managing interfacial electrochemistry. Acc. Chem. Res. **40**(11), 1165-1173 (2007). <https://doi.org/10.1021/ar7000952>
4. P. K. Gupta, S. S. Raghunath, D. V. Prasanna, P. Venkat, V. Shree et al., An update on overview of cellulose, its structure and applications. Cellulose **201**(9), 84727 (2019). <https://doi.org/10.5772/intechopen.84727>
5. X. Li, H. Li, Z. Ling, D. Xu, T. You et al., Room-temperature superbase-derived ionic liquids with facile synthesis and low viscosity: powerful solvents for cellulose dissolution by destroying the cellulose aggregate structure. Macromolecules **53**(9), 3284-3295 (2020). <https://doi.org/10.1021/acs.macromol.0c00592>
6. A. Xu, Y. Zhang, Y. Zhao, J. Wang, Cellulose dissolution at ambient temperature: role of preferential solvation of cations of ionic liquids by a cosolvent. Carbohydr. Polym. **92**(1), 540-544 (2013). <https://doi.org/10.1016/j.carbpol.2012.09.028>
7. L. Zhang, Z. Jiang, S. Yang, Z. Zeng, W. Zhang et al., Different rheological behaviours of cellulose/tetrabutylammonium acetate/dimethyl sulfoxide/water mixtures. Cellulose **27**(14), 7967-7978 (2020). <https://doi.org/10.1007/s10570-020-03363-8>
8. W. Yue, L. Zhang, L. Deng, Y. Guo, Q. Xu et al., Co-dissolution of cellulose and silk fibroin in levulinic acid-derived protic ionic liquids for composited membrane and fiber preparation. Green Chem. **23**(23), 9669-9682 (2021). <https://doi.org/10.1039/D1GC02837E>
9. F. Lu, J. Song, B. W. Cheng, X. J. Ji, L. J. Wang, Viscoelasticity and rheology in the regimes from dilute to concentrated in cellulose 1-ethyl-3-methylimidazolium acetate solutions. Cellulose **20**(3), 1343-1352 (2013). <https://doi.org/10.1007/s10570-013-9885-7>
10. D. C. Malaspina, J. Faraudo, Molecular insight into the wetting behavior and amphiphilic character of cellulose nanocrystals. Adv. Colloid Interface Sci. **267**, 15-25 (2019). <https://doi.org/10.1016/j.cis.2019.02.003>
11. D. Zhao, C. Chen, Q. Zhang, W. Chen, S. Liu et al., High performance, flexible, solid-state supercapacitors based on a renewable and biodegradable mesoporous cellulose membrane. Adv. Energy Mater. **7**(18), 1700739 (2017). [https://doi.org/10.1002/aenm.201700739](https://doi.org/https://doi.org/10.1002/aenm.201700739)
12. H. Wu, J. Mu, Y. Xu, F. Xu, S. Ramaswamy et al., Heat-resistant, robust, and hydrophilic separators based on regenerated cellulose for advanced supercapacitors. Small **19**(1), 2205152 (2023). [https://doi.org/10.1002/smll.202205152](https://doi.org/https://doi.org/10.1002/smll.202205152)
13. Q. Zhang, C. Chen, W. Chen, G. Pastel, X. Guo et al., Nanocellulose-enabled, all-nanofiber, high-performance supercapacitor. ACS Appl. Mater. Interfaces **11**(6), 5919-5927 (2019). <https://doi.org/10.1021/acsami.8b17414>
14. M. Hou, M. Xu, Y. Hu, B. Li, Nanocellulose incorporated graphene/polypyrrole film with a sandwich-like architecture for preparing flexible supercapacitor electrodes. Electrochim. Acta **313,** 245-254 (2019). [https://doi.org/10.1016/j.electacta.2019.05.037](https://doi.org/https://doi.org/10.1016/j.electacta.2019.05.037)
15. B. Dyatkin, V. Presser, M. Heon, M. R. Lukatskaya, M. Beidaghi et al., Development of a green supercapacitor composed entirely of environmentally friendly materials. ChemSusChem **6**(12), 2269-2280 (2013). [https://doi.org/10.1002/cssc.201300852](https://doi.org/https://doi.org/10.1002/cssc.201300852)
